# A new profiling approach for DNA sequences based on the nucleotides' physicochemical features for accurate analysis of SARS-CoV-2 genomes

**DOI:** 10.1186/s12864-023-09373-7

**Published:** 2023-05-18

**Authors:** Saeedeh Akbari Rokn Abadi, Amirhossein Mohammadi, Somayyeh Koohi

**Affiliations:** Department of Computer Engineering, Sharif University of Technology, Tehran, Iran

**Keywords:** SARS-CoV-2, Classification, Comparison, k-mer, Alignment-free

## Abstract

**Background:**

The prevalence of the COVID-19 disease in recent years and its widespread impact on mortality, as well as various aspects of life around the world, has made it important to study this disease and its viral cause. However, very long sequences of this virus increase the processing time, complexity of calculation, and memory consumption required by the available tools to compare and analyze the sequences.

**Results:**

We present a new encoding method, named PC-mer, based on the k-mer and physic-chemical properties of nucleotides. This method minimizes the size of encoded data by around 2^ k^ times compared to the classical k-mer based profiling method. Moreover, using PC-mer, we designed two tools: 1) a machine-learning-based classification tool for coronavirus family members with the ability to recive input sequences from the NCBI database, and 2) an alignment-free computational comparison tool for calculating dissimilarity scores between coronaviruses at the genus and species levels.

**Conclusions:**

PC-mer achieves 100% accuracy despite the use of very simple classification algorithms based on Machine Learning. Assuming dynamic programming-based pairwise alignment as the ground truth approach, we achieved a degree of convergence of more than 98% for coronavirus genus-level sequences and 93% for SARS-CoV-2 sequences using PC-mer in the alignment-free classification method. This outperformance of PC-mer suggests that it can serve as a replacement for alignment-based approaches in certain sequence analysis applications that rely on similarity/dissimilarity scores, such as searching sequences, comparing sequences, and certain types of phylogenetic analysis methods that are based on sequence comparison.

**Supplementary Information:**

The online version contains supplementary material available at 10.1186/s12864-023-09373-7.

## Background

In 2019, a new virus has emerged called SARS-CoV-2 (severe acute respiratory syndrome coronavirus-2), which causes severe illness. The World Health Organization (WHO) declared this disease, known as COVID-19 (coronavirus disease 2019), a global pandemic on March 11, 2020 [1]. COVID-19 has now spread to over 184 countries and has infected more than 400 million individuals. Coronaviruses are enveloped, linear, positive-sense, and single-stranded RNA viruses, and have the largest viral genomes among all RNA viruses, measuring around 30 kb [1, 2]. During a virus outbreak, understanding the taxonomic classification of apathogenic and specied and its relationship to other pathogens can help with the development of appropriate mitigation strategies, containment, treatment, and decisions on the methods and measures appropriate for identifying viruses, controlling their transmission rates, and limiting potential consequences [2–4].

The early identification of SARS-CoV-2 as a close relative of MERS-CoV and SARS could potentially benefit global efforts to design and develop a vaccines and therapeutic drugs for SARS-CoV-2. This is because it would lead to an improved understanding of the disease progression, host–pathogen interactions, and potential treatment strategies [2]. However, the classification of SARS-CoV-2 is not a simple task due to its significant genetic similarities to other Coronaviridae viruses. This could result in incorrect findings and false positives [4]. Coronavirus genomes, like other RNA viruses, are known for their genomic plasticity, which can be attributed to factors such as high mutation rates ranging from 1 in 1000 to 1 in 10,000 nucleotides during replication, the use of a template-switching mechanism that results in high rates of homologous RNA recombination between viral genomes, and a large genome size that leads to a high incidence of mutation. As a result,the genomes of coronavirus today are more flexible and varied [3]. SARS-CoV-2 has been classified using various methodologies, including both alignment-based and alignment-free categories, which is similar to the methods used for other species [2, 3]. To achieve this classification, many types of data have been analyzed, such as whole genomes, partial genomes, protein data, and even medical images, like CT scans of the lungs [1, 3, 5]. For instance, research conducted by comparing whole genome and viral proteins has shown that SARS-CoV-2 belongs to lineage B (Sarbecovirus) of Betacoronavirus. Furthermore, through the phylogenetic investigation of the RdRp protein, spike proteins, and complete genomes, a close relationship between SARS-CoV-2 and two bat SARS-like coronaviruses, bat-SL-COVZXC21 and bat-SL-COVZC45, identified in Chinese horseshoe bats Rhinolophus sinicus, has been revealed. These findings, as well as others, have been examined in numerous studies [3, 5–7], which mostly adopt alignment-based methods. However, while alignment-based approaches have proved effective in detecting sequence similarities, their application can be challenging in certain circumstances [3, 4]. With the improvement of sequencing technology and an increase in the number of sequenced genomes, the usage of alignment-free approaches for sequence comparison has been on the rise [2–4, 7].

An alignment-free approach [8] is a method of measuring sequence similarity or dissimilarity without using or producing aligned sequences during any stage of the algorithm implementation. This approach is faster, uses fewer resources, and is more robust to structural variation compared to alignment-based approaches. Additionally, alignment-free methods can be used in cases where alignment cannot safely handle low sequence conservation. These benefits make alignment-free approaches useful for comparing and categorizing viruses. Various alignment-free methods have been developed specifically for SARS-CoV-2 in recent years. For instance, authors in [3] utilized the MLDSP approach [9] to classify SARS-CoV-2, which was developed earlier for other species. MLDSP combines supervised machine learning and FCGR (Frequency of Chaos Game Representation) methods. FCGR representation [7, 8, 10] is a well-known encoding approach that uses k-mers' frequencies for taxonomic classification of genomic sequences. To detect SARS-CoV-2, the researchers employed six supervised machine learning classifiers (linear discriminant, linear support vector machine, quadratic support vector machine, fine KNN (K-Nearest Neighbors), subspace discriminant, and subspace KNN). MLDSP utilizes machine learning classifiers trained with samples from four Coronaviridae families and tests them on 29 SARS-CoV-2 genome sequences to identify the correct cluster [3]. However, this method has a drawback: it uses six machine learning classifiers, making it difficult to choose the best one. Additionally, in [3], samples are classified at the genus level rather than the more crucial and challenging species or inner-species level. Despite this weakness, [3] is one of the most significant and attractive SARS-CoV-2 approaches to date.

GSP [11] proposes two approaches for distinguishing SARS-CoV-2, SARS-CoV, and MERS-CoV viruses, both based on K-nearest neighbors and trainable cascade forward back-propagation neural networks, making it another method for classifying SARS-CoV-2. It uses a discrete Fourier transform (DFT), a discrete cosine transform (DCT), and the seven-moment invariants to extract genomic signal processing features from each sequence. GSP [11] utilizes a dataset of 76 genome sequences for each coronavirus type. The simulation results indicate that the KNN algorithm performs better in all SARS-CoV-2/SARS-CoV, SARS-CoV-2/MERS-CoV, and SARS-CoV-2/SARS-CoV/MERS-CoV classification processes, with 100% accuracy. One of the method's key advantages is the limited number of extraction attributes recovered by each approach, just nine for each sequence. However, like MLDSP [3] and GSP [11], the classification capability of SARS-CoV-2 using a small number of distinct and easily classifiable clusters is limited. To overcome these concerns, various classification approaches for SARS-CoV-2 are continuously being developed. For instance, in [2], several assessments and methods are proposed for performing classification both between species (SARS-CoV-2, MERS-CoV, Dengue Virus (DENV), Zaire Ebolavirus (EBOV), Hepatitis B virus (HBV), Hepatitis C virus (HCV), Human Immunodeficiency Virus 1 (HIV-1), and Mycobacterium tuberculosis (M. tb)) and within species (SARS-CoV-2 from Africa, Asia, Europe, Oceania, North America, and South America). These methods aim to enhance the classification accuracy of SARS-CoV-2 and provide a more comprehensive understanding of its phylogenetic relationships with other viruses. The dataset used in this study is distinguishable and can be easily classified. The encoding method used is dinucleotide frequency, one of the most commonly used methods, and various assessments were conducted, including principal component analysis (PCA) and t-distributed Stochastic Neighbor Embedding (t-SNE), to analyze the datasets by reducing their dimensionality. The study utilized an unsupervised learning approach through agglomerative hierarchical clustering, multi-class classification, and their binarized forms. In addition to reporting classification accuracy, this study mainly analyzed the frequency of different dinucleotides in different categories, examining the potential of dinucleotides as species signatures [2].

Most classification approaches proposed for coronaviruses [1–3, 7, 12], as well as other viruses like HIV and Influenza A [13] and other species such as bacteria or mammals [14, 15], rely on using the frequencies of k-mers as a distinguishing feature of sequences. After reviewing various studies in this area, it can be concluded that the size of k-mer is directly proportional to the accuracy of classification [3, 16]. However, accuracy alone cannot be considered a conclusive metric, and it is necessary to report other metrics like precision and F1-score, although they are often overlooked in many studies [3]. Additionally, the memory usage increases exponentially with each unit rise in k-mer size, leading to four times more memory usage. Naturally, there have been other limitations noted in studies of the tools created for SARS-CoV-2 comparison and clustering. These limitations include difficulties distinguishing between closely related virus strains, as well as lower resolution due to the exclusion of genomes with high similarity. Additionally, some tools are unable to detect multiple strains in the case of multi-strain infections [7, 17]. Given the limitations of current classification methods, we present an approach in this study to reduce k-mer encoding's memory usage while improving accuracy and other performance metrics. However, reducing memory usage and enhancing the speed and precision of machine learning-based classifiers is not the only aim. The nature of the data used and the level of evolution are also crucial factors to consider. In this work, we introduce a novel encoding method called PC-mer (PhysicoChemical k-mer-based encoding), which utilizes both the FCGR method and the physicochemical properties of nucleotides to reduce k-mer memory size. The proposed encoding method is extremely effective, enabling even the simplest machine learning-based classifier to accurately classify input sequences across various levels of evolution. We have extensively tested this tool on several popular datasets, including the MLDSP dataset, as well as datasets with high similarity between clusters, such as [1]. The following are the primary advantages of PC-mer:

- The amount of memory usage for storing k-mers is reduced by approximately 2^ K^.
- Classification accuracy is improved, compared to other classifiers such as MLDSP, utilizing a very simple machine learning-based classifier.
- Training of the machine learning-based classifier is accelerated.
- No prior knowledge of input data is required.
- The proposed classifier, taking advantages of PC-mer, achieves high accuracy on a simple personal computer.

## Results

As discussed earlier, our new encoding method, PC-mer (PhysicoChemical k-mer-based encoding), reduces the memory size of k-mers by combining the FCGR method with the physicochemical properties of nucleotides. This encoding method is so powerful that even the simplest machine learning-based classifier can classify input sequences accurately at different levels of evolution. In this section, we evaluate the performance of the PC-mer encoding method in two ways: 1) using PC-mer as the input data generator for classification models, and 2) applying PCA (Principal Component Analysis) to make the encoded data by PC-mer more interpretable, thereby evaluating PC-mer's ability to distinguish different classes of input sequences.

### Performance evaluation

The classification results are presented for four different datasets:

1. SARS-CoV-2 datasets consisting of seven different levels of taxonomy, namely Test-1, Test-2, Test-3a, Test-3b, Test-4, Test-5, and Test-6. At lower levels, the datasets become more similar to each other.
2. Human coronavirus (Corona) datasets, collected from human samples, which are more challenging to classify compared to SARS-CoV-2 datasets.
3. 35 sequences of Human coronavirus used to compare the alignment-free approach based on PC-mer with a well-known alignment-based method.
4. 45 SARS-CoV-2 sequences used to compare the alignment-free approach based on PC-mer with a well-known alignment-based method for very similar sequences.

### ML-based classification performance

For the machine learning-based evaluations, we utilized a tenfold cross-validation scheme and assessed the results based on commonly used statistical measures, such as accuracy, precision, recall, and F1-score, which are presented in Tables 1 and 2. As shown in Table 1, we achieved 97% accuracy using PC-mer encoding and a linear SVM model for the SARS-CoV-2 dataset and Test-1, which is the first level of taxonomy consisting of 3273 sequences divided into 12 clusters (11 families, and Riboviria realm). In this evaluation, we used the PC-mer encoding method as input data. Additionally, compared to the FCGR representation and the best classification model (Quadratic SVM) used by the MLDSP-GUI tool, the proposed encoding method results in a 2% increase in classification accuracy. Additionally, after training the model on the Test-1 dataset, we validated and tested the resulting classifier to predict the labels of 29 SARS-CoV-2 virus sequences, achieving a 100% classification accuracy for the label Riboviria. Moving on to the second dataset (Test-2), which consists of 2777 sequences divided into 12 clusters (Riboviria families), we obtained a classification accuracy of 95.9% using the PC-mer encoding and the Linear SVM classifier. In this dataset, we observed a 4.7% improvement in classification accuracy compared to the best classification model (Linear Discriminant) and FCGR representation used by the MLDSP-GUI tool. Similarly, after training the model on Test-2 dataset, we validated and tested the resulting classifier to predict the labels of 29 SARS-CoV-2 virus sequences, achieving a 100% classification accuracy for the label Coronaviridae.

**Table I:** Investigating the encoding capability of the PC-mer (using only Linear SVM classifier) vs. that of the FCGR (using six classifiers: Linear Discriminant (LD), Linear SVM (LSVM), Quadratic SVM (QSVM), Fine KNN (FKNN), Subspace Discriminant (SD), and Subspace KNN (SKNN)) as the encoding methods for generating input vectors

*Encoding algorithm*	*k-mer*	*Classification model*	*Metrics*	*Datasets (%)*							
				***Test-1***	***Test-2***	***Test-3a***	***Test-3b***	***Test-4***	***Test-5***	***Test-6***	***Human Coronavirus***
PC-mer	12	Linear SVM	Accuracy	**97.25**	**95.93**	**98.52**	**100**	**100**	**99.33**	**100**	**100**
			F1	97.23	95.9	98.49	100	100	99.36	**100**	**100**
			Precision	97.38	96.16	98.85	100	100	99.55	**100**	**100**
			Recall	97.25	95.93	98.52	100	100	99.33	**100**	**100**
FCGR	7	LD	Accuracy	91.7	91.2	98.1	**100**	97.6	98.6	**100**	-
		LSVM		90.8	89.2	94.2	93.3	98.4	97.4	**100**	-
		QSVM		95	93.1	95.2	93.3	98.4	97.4	**100**	-
		FKNN		93.4	90.3	95.7	95	97.6	97.4	**100**	-
		SD		87.6	89	97.6	95	98.4	98.7	**100**	-
		SKNN		93.2	90.4	96.2	95	97.2	96.1	**100**	-
		Average accuracy		92	90.5	96.2	95.3	97.6	97.5	**100**	-

**Table 2:** Prediction accuracy of PC-mer for predicting SARS-CoV-2 label in various taxonomic levels

Training dataset	Testing dataset	Prediction accuracy	Predicted label
Test-1	29 SARS-CoV-2 virus sequences	100%	Riboviria
Test-2	29 SARS-CoV-2 virus sequences	100%	Coronaviridae
Test-3(a\b)	29 SARS-CoV-2 virus sequences	100%	Betacoronavirus

For the third dataset (Test-3a), which consists of 208 sequences divided into 4 classes (Coronaviridae), we obtained 98.5% classification accuracy using the PC-mer encoding and Linear SVM model, slightly higher than the best classification model (Linear Discriminant) used by the MLDSP-GUI tool. Additionally, the trained Linear SVM model successfully predicted the label Betacoronavirus for all 29 SARS-CoV-2 virus sequences, achieving 100% classification accuracy. As for the fourth dataset (Test-3b), which comprises 60 sequences grouped into 3 clusters (Coronaviridae), we achieved 100% classification accuracy using PC-mer encoding and Linear SVM model, similar to the accuracy achieved by the best classification model (Linear Discriminant) and FCGR representation used by the MLDSP-GUI tool [9]. Finally, the trained Linear SVM model successfully predicted the label Betacoronavirus for all 29 SARS-CoV-2 virus sequences, resulting in 100% classification accuracy. Moving on to the fifth dataset (Test-4), which consists of 124 sequences divided into 4 clusters (Betacoronavirus), we achieved 100% classification accuracy using the PC-mer encoding and Linear SVM model. Therefore, our proposed encoding method led to a nearly 2% increase in classification accuracy compared to the best classification model (Linear Discriminant) and FCGR representation used by the MLDSP-GUI tool. Similarly, for the 29 SARS-CoV-2 virus sequences in this dataset, the trained Linear SVM model predicted the label Sarbecovirus for all 29 sequences, achieving 100% classification accuracy. For the sixth dataset (Test-5), which includes 153 sequences divided into 5 clusters of Betacoronavirus, excluding SARS-CoV-2 and SARS-CoV-2 virus, we achieved a classification accuracy of 99.3% using the PC-mer encoding and Linear SVM classifier. With this dataset, we were able to improve the classification accuracy by nearly 1% when compared to the best classification model (SubspaceDiscriminant) and FCGR representation used by the MLDSP-GUI tool [9].

**Fig. 1:**
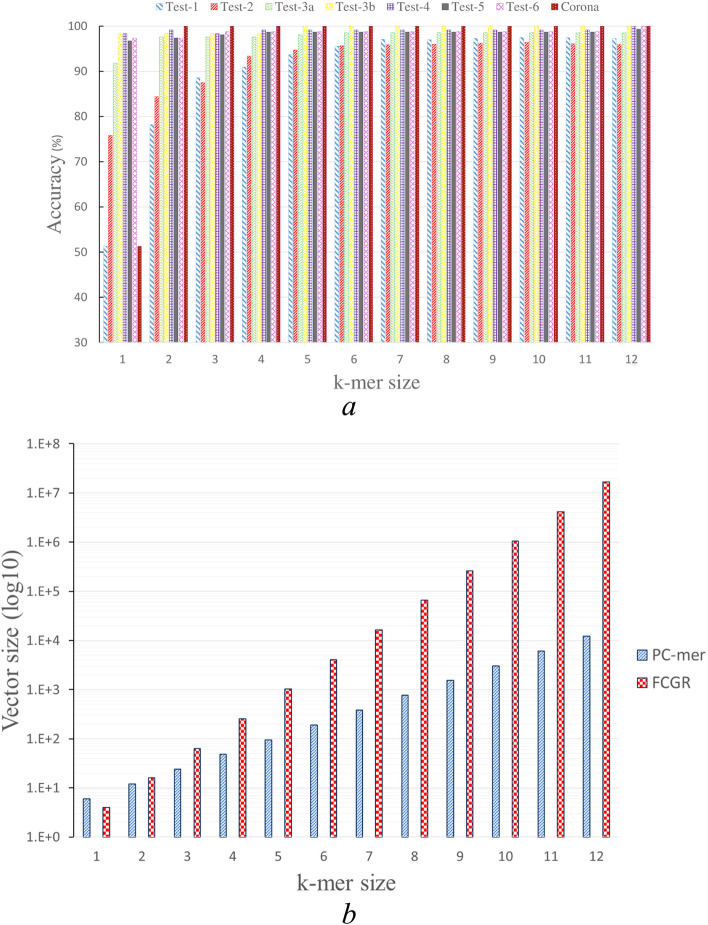
**a** Accuracy of ML-based classifier based on PC-mer for various k-mer sizes, b) the number of required storage units for PC-mer and FCGR matrices for various values of k

**Fig. 2:**
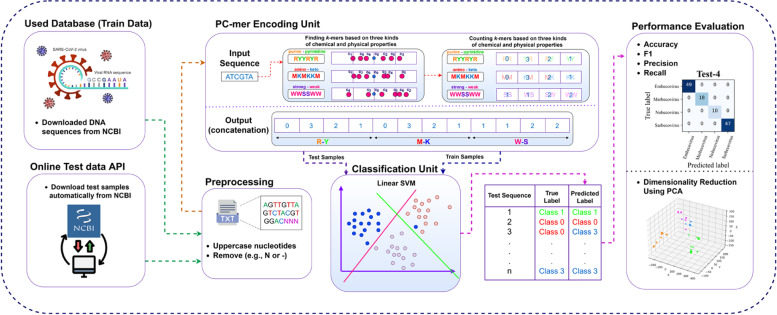
PC-mer method's pipeline

On the other hand, in the seventh dataset (Test-6), which represents the last level of taxonomy and contains 76 sequences divided into 2 clusters (Sarbecovirus and SARS-CoV-2 virus), we achieved 100% classification accuracy using PC-mer encoding, which is similar to the results obtained by all other classification models and the FCGR representation used by the MLDSP-GUI tool. For the latest dataset (coronavirus), which includes 874 human coronavirus sequences divided into 7 clusters, we achieved 100% classification accuracy using PC-mer encoding and the Linear SVM model. We evaluated the performance of the proposed encoding method by its feature extraction capability and its sensitivity to the variation of k-mer size in the range of [1, 16] (see Table S 4 in the supplementary materials). While more detailed results are available in the supplementary materials, Table 1 summarizes the classification accuracies for a k-mer size of 12, for the sake of brevity. It is worth noting that PC-mer encoding enables the use of larger k-mers by reducing the size of the encoded data. The proposed encoding is designed to reduce the computational complexity of k-mer extraction and the size of the generated data from $O\left({4}^{k}\right)$ to $O\left({2}^{k}\right)$. For instance, the FCGR approach, assuming k = 7, produces a vector of size 16,384 for each genome sequence, while the PC-mer encoding method, assuming k = 12, produces a vector of size 12,288 for each genomic sequence. This is significantly less than the size of the data generated by the FCGR approach for k = 7. It should be noted that using the FCGR approach and assuming k-mers of size 12 results in an encoded vector with a size of 16,777,216 for each genome sequence. According to the main benefits of the proposed encoding method, PC-mer generates small vectors of size 384 for each genome sequence with k = 7, as opposed to the large vectors generated by the MLDSP tool for k-mers of size 7. Therefore, it can be concluded that the data compression ability of PC-mer significantly reduces runtimes of preprocessing, training, and testing phases while also improving classification accuracy and enabling the use of larger k-mers. These advantages are presented in Fig. 1, and all experiments were conducted on a standard desktop computer. It should be noted that our pipeline, as shown in Fig. 2, includes an online data API that allows any standard dataset to be downloaded from NCBI and automatically encoded by PC-mer. Consequently, the encoded data can be utilized for training or testing any arbitrary classifier.

### Evaluating computational comparison

To evaluate the performance of PC-mer in comparison to other computational methods, we utilized the proposed method with a k-mer size of 12 on two additional datasets. Next, we calculated the Manhattan distance between the PC-mer vectors corresponding to each sequence pair, resulting in a distance matrix for each dataset. We then used five alignment-free methods (BBC, NCD, wmetric, word-bool, and word-sets) available in the ALFPY tool [8] to comprehensively evaluate and compare PC-mer with well-known alternative methods on the human coronavirus and SARS-CoV-2 datasets. For the evaluation of word-bool and word-sets, we assumed a k-mer size of 7, as mentioned earlier. As a reference comparison result, we performed a global alignment between the two sequences using the pairwise alignment function (Smith-Waterman algorithm) in R with the default configuration, generating another distance matrix. Detailed results can be found in the supplementary materials. Finally, to conduct the comparative study, we computed the correlation coefficient between the six distance matrices generated by PC-mer, BBC, NCD, wmetric, word-bool, and word-sets, and the reference matrix generated by the Smith-Waterman algorithm, as presented in Table 3. It is important to note that the pairwise alignment function is used as a benchmark alignment-based method for sequence comparison [8], and its output is considered as the reference result. After performing the various steps of the analysis, we obtained correlation coefficient values of 98.08% and 93.75% for the human dataset and SARS-CoV-2 dataset, respectively. These results demonstrate that, in addition to its usefulness in ML-based comparison approaches, the PC-mer encoding method can also be used by computational algorithms for sequence comparison.

**Table 3:** Correlation coefficient between distance matrix obtained from six compared methods PC-mer, CLUSTALW, BBC, NCD, Wmetric, Word-bool, Word-sets with reference Smith-Waterman method

Methods		PC-mer	BBC	NCD	Wmetric	Word-bool	Word-sets
**Correlation coefficient**	Human coronaviruses dataset	0.9808	0.9490	0.9463	0.6738	0.9796	0.9797
	SARS-CoV-2 dataset	0.9374	0.7860	0.8847	0.7046	0.9297	0.9292

### Analyzing the Discriminability of the PC-mer Coding Using PCA

**Fig. 3:**
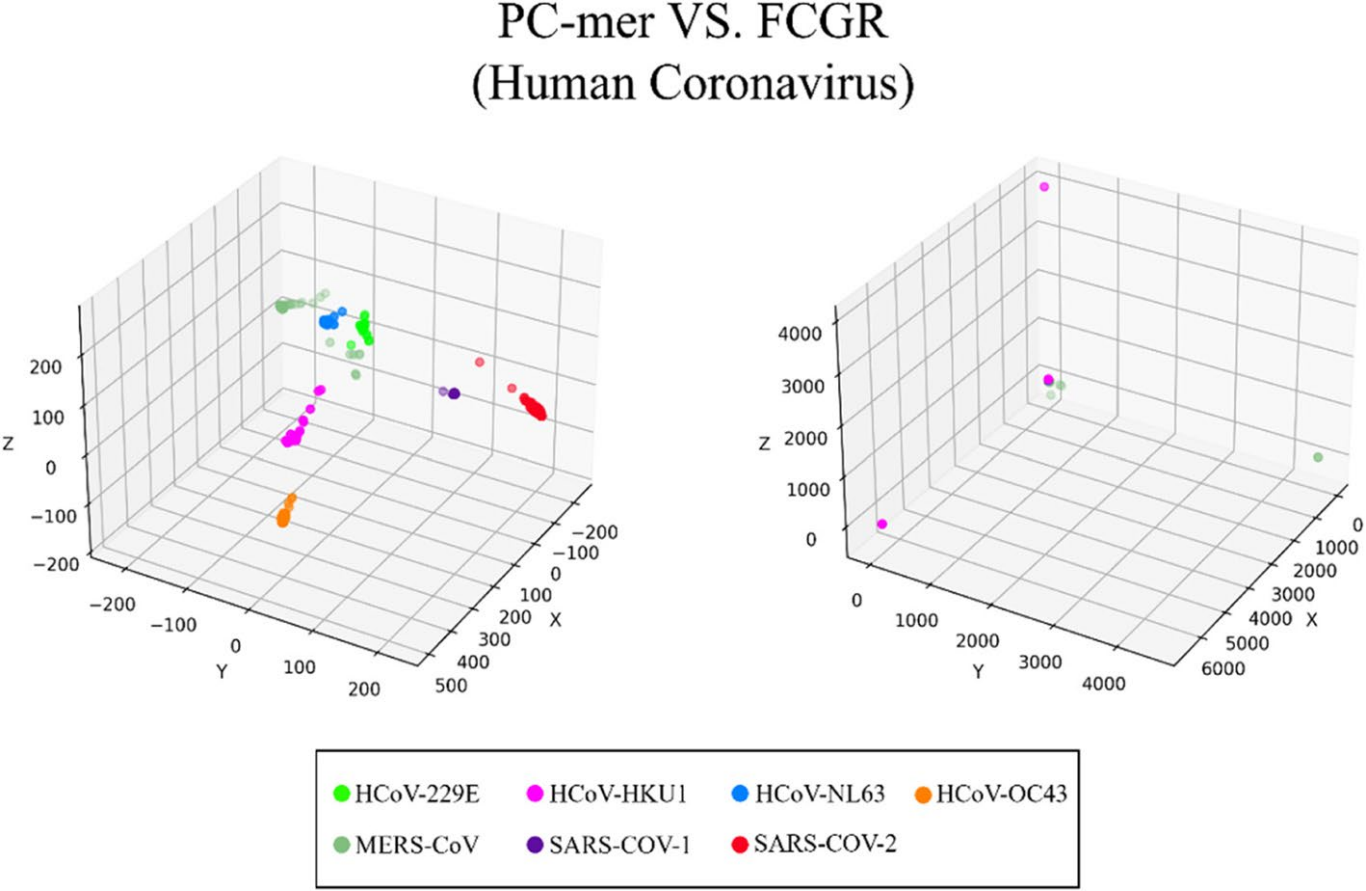
PCA of PC-mer and FCGR for Human coronaviruses datasets

**Fig. 4:**
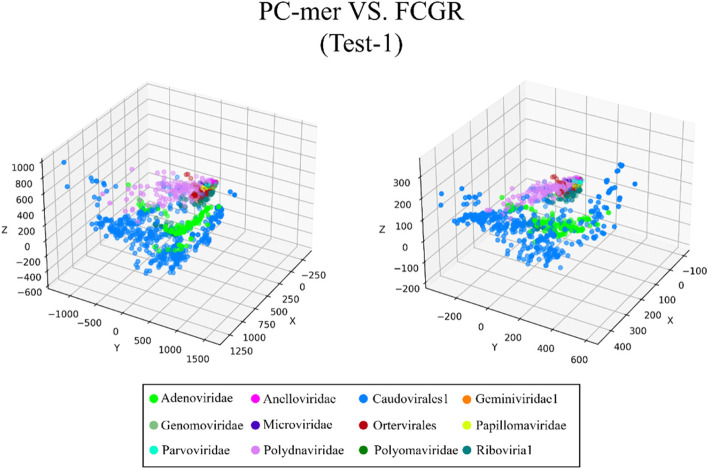
PCA of PC-mer and FCGR for test-1 datasets

**Fig. 5:**
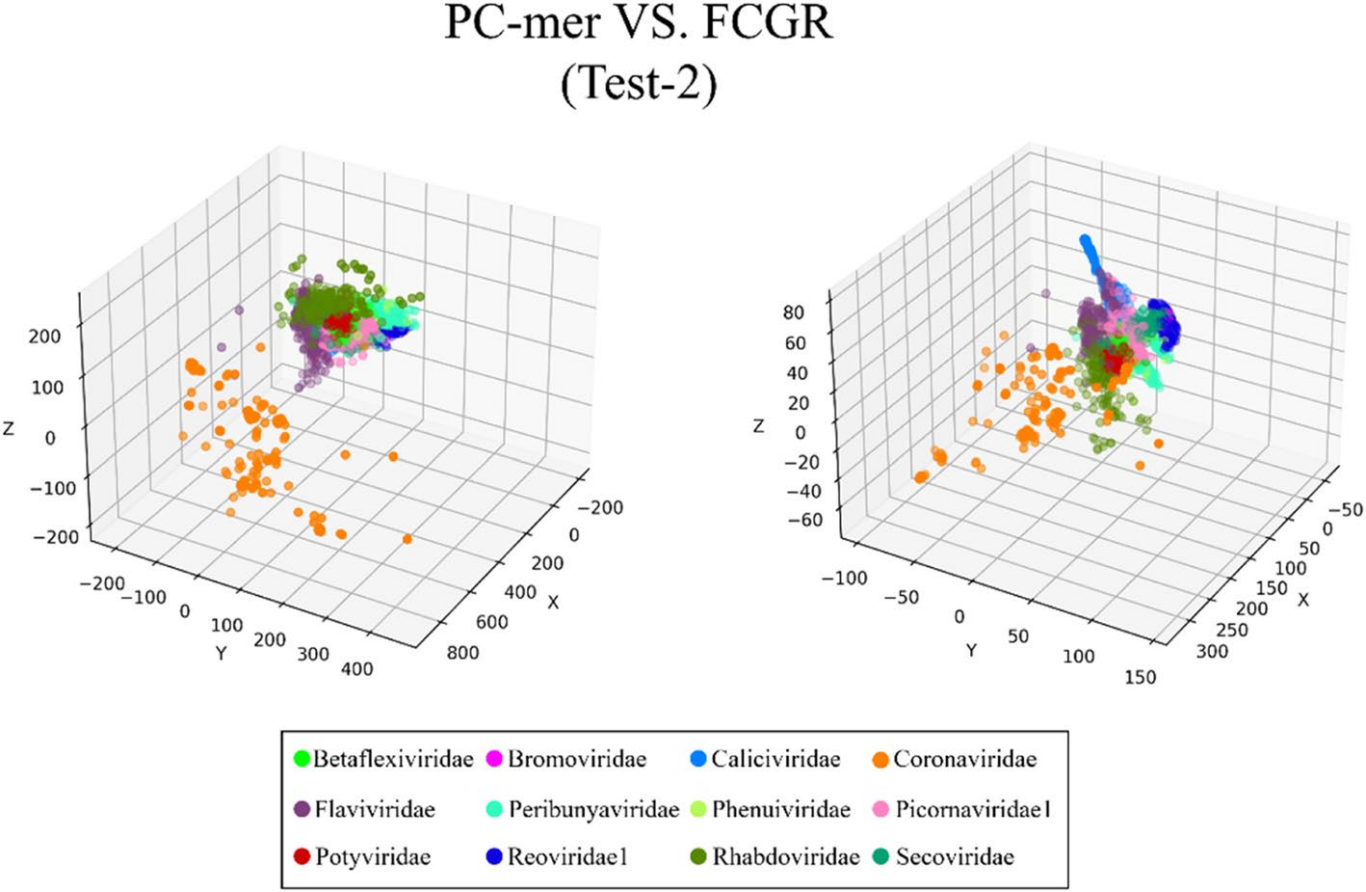
PCA of PC-mer and FCGR for test-2 datasets

**Fig. 6:**
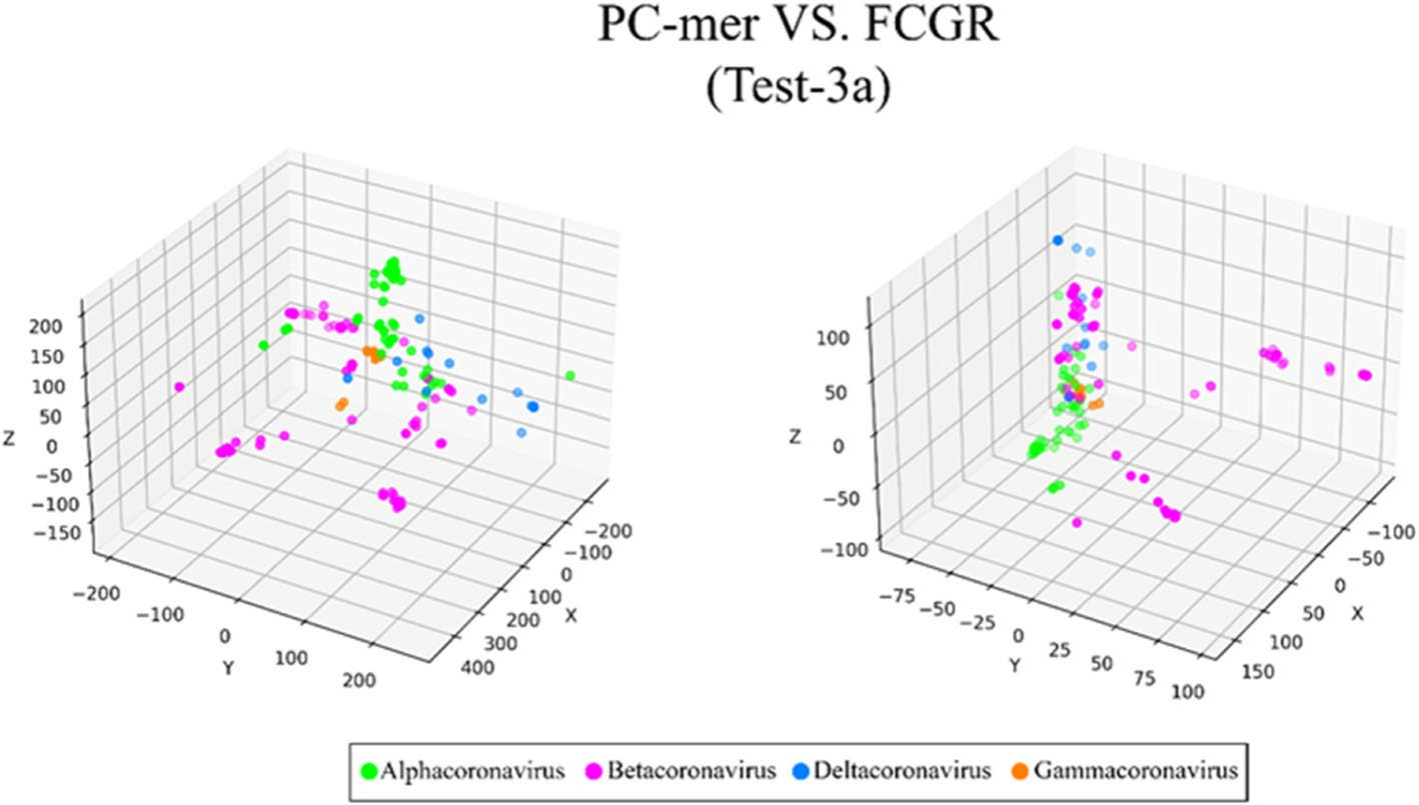
PCA of PC-mer and FCGR for test-3a

**Fig. 7:**
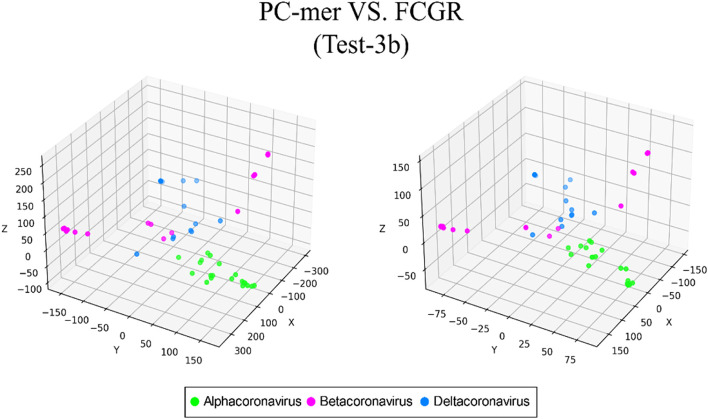
PCA of PC-mer and FCGR for test-3b

**Fig. 8:**
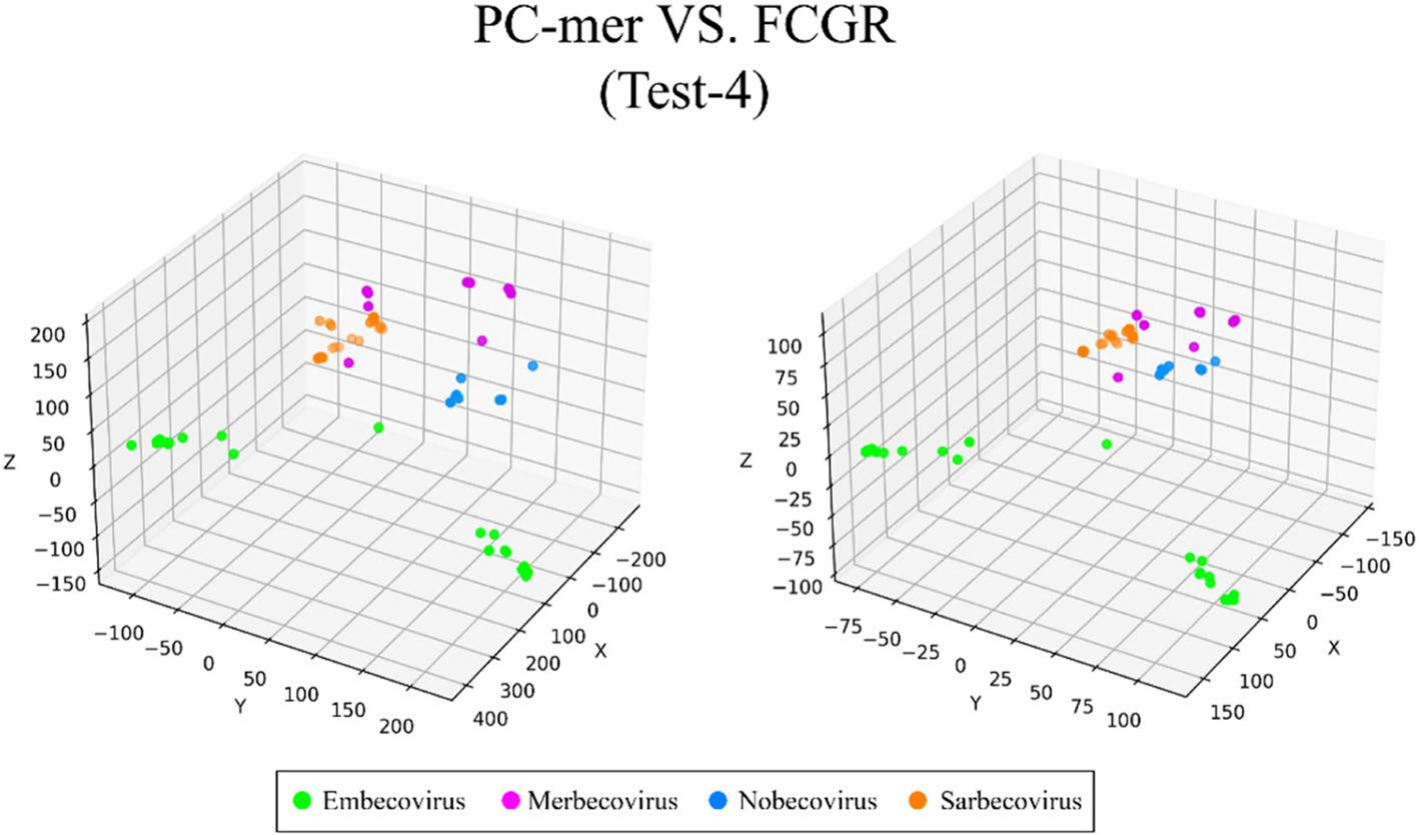
PCA of PC-mer and FCGR for test-4 dataset

**Fig. 9:**
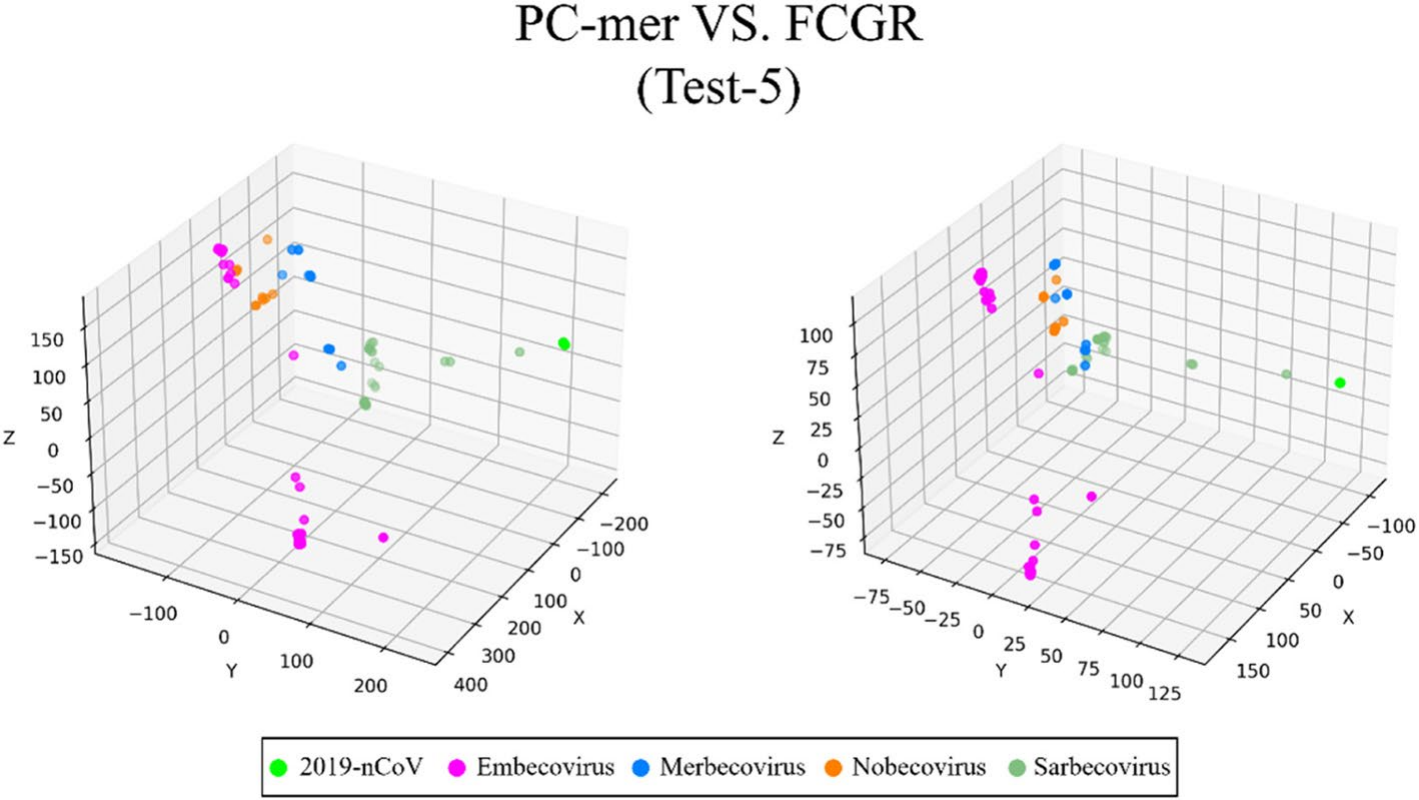
PCA of PC-mer and FCGR for test-5 dataset

**Fig. 10:**
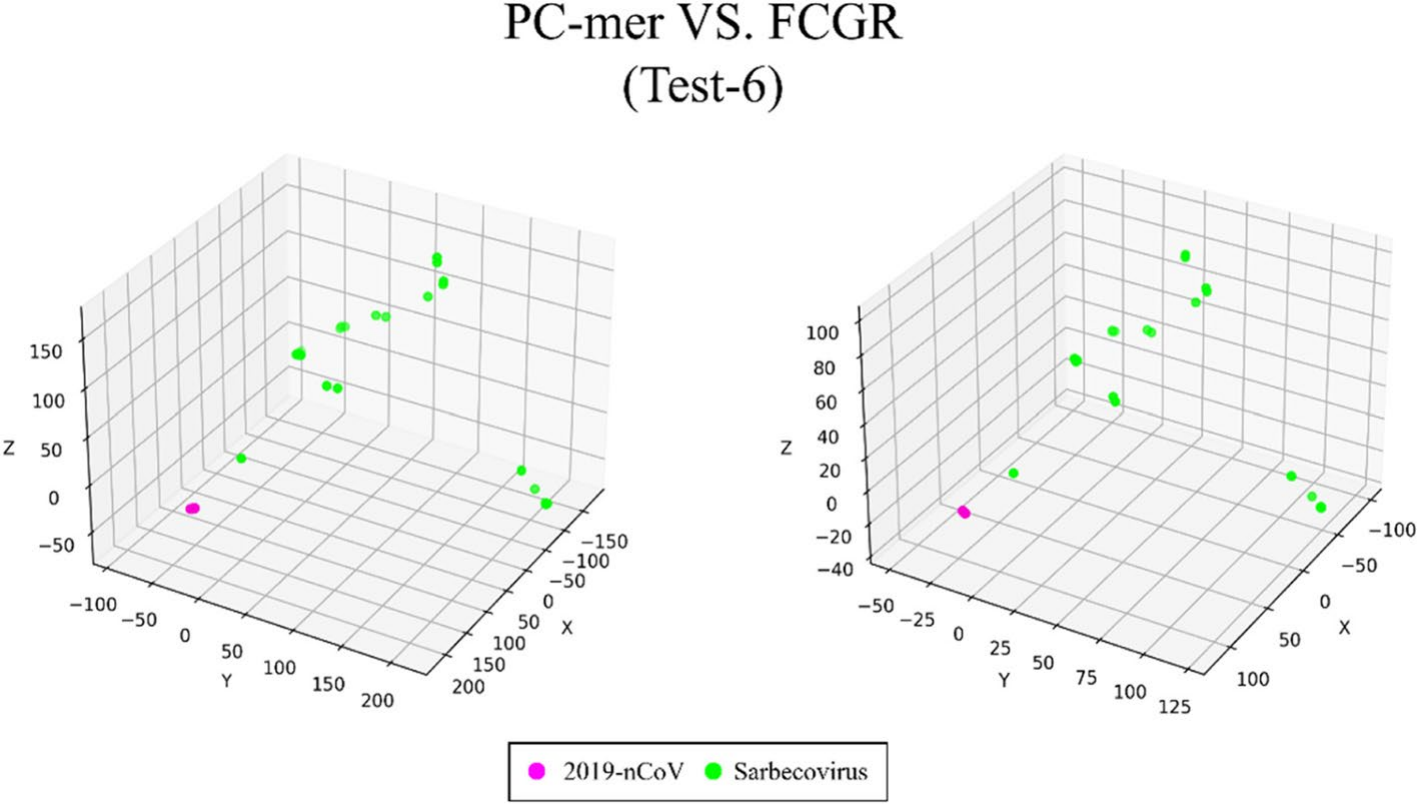
PCA of PC-mer and FCGR for test-6 dataset

The ability to distinguish between different classes of input sequences is a fundamental aspect of any encoding method, and its importance cannot be overstated. Discriminatory encoding methods allow for the use of a simple classification algorithm to identify the optimal boundary between distinct clusters, thereby reducing computational costs and preprocessing time required to select the appropriate classification algorithm. Additionally, the proper discrimination of encoded data facilitates the use of dimensionality reduction algorithms that extract global features and reduce the size of input data. As large datasets are increasingly common across many disciplines, it is essential to employ methods that significantly reduce their dimensionality in a meaningful way while retaining most of the information content of the original data. Although numerous techniques have been developed for this purpose, PCA is known to be one of the oldest and most widely used methods. It reduces the dimensionality of a dataset while preserving as much statistical information (i.e., variability) as possible. Therefore, we conducted an experiment to evaluate the discriminability of the PC-mer encoding method after significant dimensionality reduction using PCA. To visually represent the encoded data by the PC-mer method and its degree of discrimination, we reduced the dimension of the encoded data from 12,288 to 3 using the PCA algorithm, assuming a k-mer size of 12. Additionally, for a comparative study, we encoded the data using the FCGR coding, assuming a k-mer size of 7, and reduced its dimensions from 16,384 to 3 using the PCA algorithm. The comparative results confirm the superior discrimination capability of PC-mer compared to FCGR using only 3 dimensions. The three-dimensional diagrams, depicting the PCA results of the PC-mer and FCGR encoding methods, are presented in Figs. 3, 4, 5, 6, 7, 8, 9 and 10 for Corona, Test-1, Test-2, Test-3a, Test-3b, Test-4, Test-5, and Test-6 datasets, respectively. In these figures, the left-side plot displays the PCA of the PC-mer method with k = 12, while the right-side plot illustrates the PCA of the FCGR method with k = 7. Our comparison of these figures reveals that the PC-mer method can discriminate different classes of all datasets more effectively than the widely used FCGR encoding approach, using fewer features and less memory usage than FCGR. For instance, as illustrated in Fig. 4, PC-mer is able to represent the distance between various samples of Test-1 within a space that is approximately three times larger than that of FCGR in all three dimensions (x, y, and z). Similar improvements are also observed in Fig. 3 through Fig. 10. As demonstrated in these figures, PC-mer's superior discriminability between different classes leads to enhanced classification performance. It is noteworthy that this improvement is also evident in Fig. 7, 8, 9, and 10, where the samples are located similarly in three dimensions for both PC-mer and FCGR. As illustrated in these figures, PC-mer exhibits a wider separation between the samples compared to FCGR, thereby providing a better representation of their distances.

## Discussion

In late December 2019, a novel coronavirus, SARS-CoV-2, was reported in China and quickly spread worldwide. This virus is genetically similar to SARS-CoV and causes a severe illness known as COVID-19, which has resulted in a significant number of deaths worldwide [1]. However, identifying SARS-CoV-2 can be challenging since the virus shares genetic similarities with other viruses in the Coronaviridae family, making it difficult to distinguish between them [4]. This presents a significant challenge since the detection of SARS-CoV-2 may lead to false positive results due to the presence of other similar viruses [4]. Hence, it is crucial to accurately differentiate the SARS-CoV-2 virus from other similar viruses for improving patient diagnosis and managing the outbreak. This time-critical issue demands high-speed sequence comparison and classification of thousands of known sequences to narrow down the possible origin candidates. Therefore, relying on alignment-based methods, which are time-consuming and may not guarantee homologous sequence continuity, can be challenging.

On the other hand, alignment-based methods are not suitable for efficiently comparing a large number of sequences that differ widely in composition [3]. To overcome the limitations of alignment-based methods [3, 18], several alignment-free methods have been proposed as high-speed alternatives capable of handling a large number of sequences [6, 19, 20]. Furthermore, since alignment-free methods compare homologous or comparable sequences, they can provide accurate and relevant quantitative comparisons even when input sequences originate from diverse locations with varied compositions [3]. Despite these advantages, alignment-free methods like MLDSP-FCGR extract a large volume of data per sample, which is $O({4}^{k})$. Therefore, there is a trade-off between classification accuracy and computation speed. Specifically, increasing the k-mer size improves classification accuracy at the expense of reducing classification speed and dramatically increasing the volume of generated data. The latter requirement necessitates the use of powerful and specialized processing platforms, such as external servers over the Internet. However, policy, legality, and regulatory issues may prohibit the transmission of patients' sequence data to an external server. Additionally, various concerns about privacy policies and the possibility of data breaches can limit researchers' and clinicians' use of such servers. Therefore, the developed software should be made available as an offline and standalone version. Recently, many data scientists have been extensively researching the virus's remarkable features. Furthermore, artificial intelligence applications and machine learning-based methods have been successfully used to accelerate the diagnosis process and improve the classification accuracy of COVID-19 cases [3, 4]. Nevertheless, without a genomic sequence encoding method, a large volume of data is generated per genomic sequence. Therefore, an efficient encoding method can not only enhance the classification accuracy but also enable the utilization of popular machine learning-based models. Furthermore, reducing the volume of generated data, an efficient encoding method can extract large k-mers while improving processing speed on desktop computers. Consequently, as sequencing technology becomes more affordable and accessible, the computational challenges of sequence analysis become increasingly significant. This trend drives the current focus of classifier development towards faster alignment-independent solutions.

In this study, we propose an alignment-free classification method based on a machine learning model that takes advantage of a novel encoding algorithm called PC-mer. This algorithm reduces the volume of generated feature vectors from $O({4}^{k})$ to $O({2}^{k})$ per sample, resulting in a reduced amount of data for large k-mer sizes compared to the traditional FCGR method. PC-mer offers several advantages: firstly, it leads to higher classification accuracy of the Coronaviridae family using machine learning algorithms, with no prior adjustments. Secondly, it reduces memory usage by about 2^ k^ times compared to traditional k-mer based encoding techniques. Finally, our proposed compression encoding method has led to a notable increase in training speed and a significant improvement in classification accuracy for both SARS-CoV-2 and Human coronavirus datasets. As shown in Table 1, our method outperforms the reference methods [3] in terms of classification accuracy. Moreover, we have significantly reduced the false positive and false negative rates across various levels of Coronaviridae family, as demonstrated by the confusion matrices in Fig. S1 to Fig. S7 of the supplementary materials.

Based on our simulation study, when using small k-mers, PC-mer achieves comparable or even higher accuracy than the FCGR method across multiple datasets. For instance, when applying PC-mer coding and a Linear SVM model to Test-1, Test-2, Test-3a, Test-3b, and Test-4 datasets with k = 5, we achieved classification accuracies of 94%, 95%, 98%, 100%, and 99%, respectively, as well as 100% classification accuracy for 29 SARS-CoV-2 virus sequences in the test data (see supplemental file "PC-mer-SM" section "Investigating the impact of size k in the PC-mer encoding method"). It is worth noting that the MLDSP tool achieved 93%, 91%, 98%, 100%, 98%, and 99% classification accuracy for the corresponding datasets, respectively, assuming k = 7 as the best k-mer length for the FCGR encoding method and various classifiers. To highlight the compression efficiency of PC-mer, it should be mentioned that the amount of data extracted per sample by the MLDSP tool using a k-mer size of 7 is approximately 170 times greater than that of PC-mer with a k-mer size of 5. Additionally, PC-mer achieved a perfect classification accuracy of 100% with a k-mer size of 1 for the Human Coronavirus dataset obtained from human samples.

As presented in Table S 3, the length of the longest sequence in the Human coronavirus dataset is 30818, which is significantly larger than the size of the extracted data per sample using PC-mer (which is 12). Thus, the high classification accuracy achieved for various datasets, while significantly reducing the size of the extracted feature vectors per sample, confirms the effectiveness of the PC-mer encoding method in feature extraction. To further investigate this effectiveness, we conducted an experiment to evaluate the discriminability of PC-mer encoding when significant dimensionality reduction (up to 3 features) is performed using Principal Component Analysis (PCA) (as described in Sect. 2.2).

**Fig. 11:**
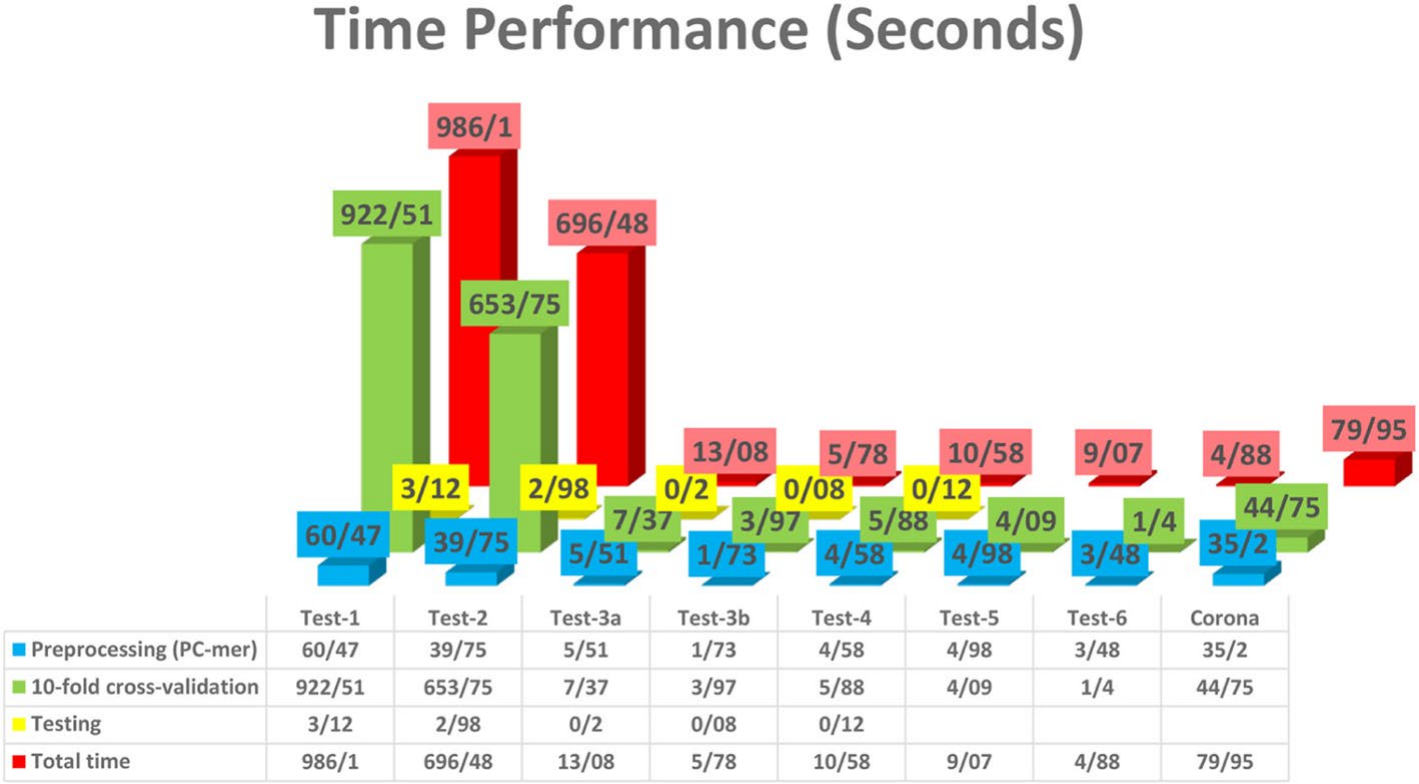
Execution times of the preprocessing, training, and testing steps of the PC-mer encoding method, assuming Linear SVM as the classification algorithm

It is noteworthy that the PC-mer encoding method has achieved superior performance without requiring any adjustments when feeding a basic machine learning algorithm, Linear SVM. In addition to its improved classification accuracy, our proposed encoding method significantly reduces the total processing time, including the preprocessing, training, and testing steps. This is due to the efficient encoding algorithm used in PC-mer, which enables the use of simple machine learning models for Coronaviridae family classification on a desktop computer with a CPU processor. Figure 11 illustrates the execution times of the preprocessing, training, and testing steps for the PC-mer encoding method when Linear SVM is used as the classification algorithm. It should be noted that this improvement in execution time can be compared to alternative approaches. Table 4 compares the PC-mer and MLDSP-GUI (FCGR) methods for various datasets used in the classification test based on machine learning. It should be noted that the processing system used for execution significantly affects the comparison results. Specifically, MLDSP-GUI (FCGR) [9] was developed on an ASUS ROG G752VS computer with 4 cores (8 threads) with a 2.7 GHz Intel Core i7 6820HK processor and 64 GB DD4 2400 MHz SDRAM, which has four times more processing cores than our system. These advanced features considered for executing MLDSP-GUI (FCGR), such as large RAMs, significantly improve the execution time. On the other hand, implementing MLDSP-GUI (FCGR) on a similar processing system to ours leads to an execution time four times longer than that of PC-mer. To ensure a fair comparison, in Table 4, we scaled down the PC-mer execution time by a factor of four. However, in reality, the reduction in PC-mer's execution time would be more than four times if the influence of RAM is also considered. As per the table, the improvements in execution time range from 20% for larger datasets to 70% for smaller datasets, with an average improvement of 44% across all datasets.

**Table 4:** Execution times (in Second) of the preprocessing, training, and testing steps of the PC-mer and MLDSP-GUI

	**Test-1**	**Test-2**	**Test-3a**	**Test-3b**	**Test-4**	**Test-5**	**Test-6**
Preprocessing (PC-mer)	15.12	9.94	1.38	0.43	1.15	1.25	0.87
Preprocessing (MLDSP-FCGR)	10.55	8.26	1.02	0.55	0.43	0.46	0.29
tenfold cross-validation (PC-mer)	230.63	163.44	1.84	0.99	1.47	1.02	0.35
tenfold cross-validation (MLDSP-FCGR)	250.01	168.63	5.11	1.95	2.04	2.4	1.85
Testing (PC-mer)	0.78	0.745	0.05	0.02	0.03	-	-
Testing (MLDSP-FCGR)	62.77	45.97	5.82	3.06	3.01	-	-
Total time (PC-mer)	246.53	174.12	3.27	1.45	2.65	2.27	1.22
Total time (MLDSP-FCGR)	323.33	222.86	11.95	5.56	5.48	2.86	2.14
Improvement by PC-mer (%)	**23.75**	**21.87**	**72.64**	**73.92**	**51.64**	**20.63**	**42.99**

In this work, we conducted a comprehensive evaluation of the PC-mer encoding method for sequence comparison using computational methods. We utilized the PC-mer encoding in an alignment-free comparison method and employed a simple computational algorithm, namely Manhattan distance calculation, to generate the distance matrix. The simulation results confirm that PC-mer enables sequence comparison and distinction at the family and species levels without the need for sequence alignment. Utilizing the advantages of PC-mer encoding, sequence comparison can be achieved rapidly with accurate comparison scores. While a simple classifier was used in this study to emphasize the encoding capability of PC-mer, it is expected that better performance can be achieved with customized classification algorithms.

It is important to note that in order to produce a dissimilarity score in the range of [0,1], the sequence dissimilarity score needs to be normalized. To achieve this, first, the PC-mer matrices are normalized by the total number of k-mers in each sequence, and then the Manhattan distances are calculated. This allows the normalized scores to evaluate the similarity of two specific sequences without the need for a large number of sequences. This achievement of the PC-mer encoding overcomes a major limitation of alignment-based methods, for which no specific range of output values exists. Therefore, evaluation of sequence similarity can only be achieved by comparing output values for a large number of sequences. Given that many k-mer-based methods are used for a variety of applications that take sequences as their input, it is expected that PC-mer will continue to be utilized in these applications as well.

## Conclusion

With confirmed cases of human-to-human transmission and concerns over asymptomatic transmission, there is an urgent need for continued intervention to prevent the spread of the SARS-CoV-2 virus. However, genome sequencing and sequence analysis in the laboratory can be both time-consuming and expensive. Furthermore, due to the high amino acid similarity between the SARS-CoV-2 virus and SARS-CoV, classifying the Coronaviridae family can be a significant challenge. In this context, computational methods using artificial neural networks, such as CNN, DLM, and GNN, can provide an efficient classification tool by leveraging hidden feature extraction. However, these methods suffer from computational overheads in terms of time and memory requirements needed to achieve high classification accuracy. Additionally, challenges such as parameter adjustment and the need to develop new classifier architectures present additional obstacles to using artificial neural networks. Therefore, encoding algorithms for genome sequences, which can be fed to classifiers, can play a crucial role in extracting hidden features, reducing computational overhead, and improving classification accuracy. Our proposed encoding method, PC-mer, is a feature extraction technique that is fast, accurate, and space-efficient. It is also compatible with a wide range of machine learning classifiers and can be used on computers with basic processing capabilities. Our simulation results, obtained by thoroughly analyzing over 5874 unique viral sequences divided into eight datasets, demonstrate the superiority of our method in terms of classification accuracy, runtime, and memory consumption. In other words, according to the comparative results presented in Sects. 2 and 3, the SVM model fed by PC-mer encoding (k = 12) outperforms MLDSP-GUI fed by FCGR encoding (k = 7), with an average execution time improvement of 44%, an accuracy improvement of more than 2%, and a 25% reduction in memory usage. Similar to k-mer-based methods, PC-mer's utility extends beyond sequence comparison, as it can be used in various computational approaches. To evaluate this capability, we analyzed a dataset with 5 samples from 7 different classes using PC-mer and the Manhattan distance, and compared the results with those obtained using a well-known alignment strategy. The simulation results showed a 98% correlation coefficient between the distance matrices produced by these two approaches. This study suggests that when rapid taxonomic classification is necessary, such as during novel viral outbreaks, an appropriate alignment-free approach for comparative genomics investigation can be crucial. To achieve this, we developed a package based on the PC-mer encoding approach, which includes a machine learning-powered classifier and a tool for computational comparison. Using simple classifiers that can also take input sequences from the NCBI database via IDs, the developed machine learning (ML)-based classifier is a fast and precise method to classify coronavirus samples into 7 clusters. In addition, the computational comparison tool in this package produces a score for every pair of input sequences as an estimate of their dissimilarity. The thorough investigation of the PC-mer technique demonstrates its potential for more comprehensive coronavirus analysis, such as examining intra-species samples and SARS-CoV-2 samples from various countries. Moreover, considering the wide range of applications that k-mer-based approaches are used for, including metagenomics classification, we will also explore the utility of PC-mer encoding in these applications in future studies. PC-mer can also be useful in certain applications that involve searching for or detecting specific sequences, such as in the identification of viruses or pathogens. In cases where the sequences of interest are highly conserved, PC-mer may be a faster and more accurate method for detecting them than alignment-based methods. This is because PC-mer is able to capture the underlying similarities between sequences without requiring them to be aligned in a specific way. Overall, while PC-mer has its limitations and is not a replacement for all types of sequence analysis, it can be a valuable tool for certain types of tasks.

## Materials and methods

### Datasets

As previously mentioned, SARS-CoV-2 datasets may include clusters from multiple taxonomic levels, with intrafamily level being a common one. The Coronaviridae family comprises four genera: Alphacoronavirus (AlphaCoV), Betacoronavirus (BetaCoV), Gammacoronavirus (GammaCoV), and Deltacoronavirus (DeltaCoV). GammaCoV and DeltaCoV primarily infect bird species, while AlphaCoV and BetaCoV infect mammalian hosts [1–3]. Various types of coronaviruses have been identified, including different AlphaCoV types such as human coronavirus 229E (229ECoV) and human coronavirus NL63 (NL63-CoV). In addition, contemporary BetaCoV types include human coronavirus HKU1 (HKU1-CoV), Severe Acute Respiratory Syndrome coronavirus (SARS-CoV), and Middle East Respiratory Syndrome coronavirus (MERS-CoV). Among them, SARS-CoV and MERS-CoV are known to cause more severe respiratory illnesses compared to other types [1]. Many studies have investigated the classification of samples from all of the aforementioned clusters, as well as other unlisted ones. Additionally, there have been efforts to distinguish SARS-CoV-2 samples from those of other clusters and correctly identify their category [1–3].

In this study, we conducted two evaluation scenarios: 1) evaluating the application of PC-mer by ML-based classifiers, and 2) evaluating the efficiency of PC-mer by computational classification methods. To assess the first scenario, we used two types of datasets to demonstrate PC-mer's ability to differentiate SARS-CoV-2 from a variety of intra/inter-species samples. One of our datasets was selected from [3] for a fair comparison with MLDSP. This dataset comprised 29 genome viruses that caused COVID-19, and was collected on January 27, 2019. Since this dataset contained viruses from different species, and the coronaviruses other than SARS-CoV-2 were selected from non-human coronaviruses, we used another dataset containing different types of human coronaviruses. We designed seven experiments at distinct taxonomic levels, each with a different number of clusters. The dataset used in this study includes 7 clusters, namely HCoV-229E, HCoV-HKU1, HCoV-NL63, HCoV-OC43, MERS-CoV, SARS-CoV-1, and SARS-CoV-2, which overcomes the limitation of the [3] dataset. Notably, this dataset has an appropriate number of samples for each cluster, which avoids the classifier being biased towards SARS-CoV-2 samples, unlike the [1] dataset. For the second evaluation scenario, we created two datasets by downloading complete sequences without any ambiguous characters. One of these datasets contains the same 7 clusters as the first evaluation scenario, with each cluster consisting of five sequences (listed in Table S 1). The second dataset includes 45 SARS-CoV-2 sequences (listed in Table S 2), which is used to demonstrate the PC-mer's ability to generate appropriate dissimilarity scores. All sequences in these datasets were retrieved from the "NCBI virus" database. More details about these datasets can be found in the "Data" section of the Supplementary materials.

### Proposed encoding method

The Frequency of Chaos Game Representation (FCGR) is a popular method for encoding sequential data, as it produces a matrix of k-mer frequencies for each sequence. To generate FCGR, the first step is to create a Chaos Game Representation (CGR) of the sequence. CGR requires a polygon with n distinct alphabets, where n represents the number of allowed alphabets in the input sequences. Unlike organizing the alphabets in a linear way, CGR provides a holistic visual representation of the sequence. The location of each letter s_i of a sequence S of length l_s on the CGR, denoted as CGR_i, is calculated by moving a pointer halfway between the position of letter ${s}_{i-1}$ in the $CGR \left(CG{R}_{i-1}\right)$ and the corner of the polygon assigned to letter $CG{R}_{{s}_{i}}$ within the CGR space, according to Eq. 1. It is important to note that the starting point of the $CGR \left(CG{R}_{0}\right)$ corresponds to the coordinates of the polygon's center point. In this study, we propose a new encoding method called PC-mer, which is an improvement over the FCGR algorithm. To achieve this, we introduce a novel categorization of the four nucleotides into three sets of structural groups based on three distinct chemical and physical features [21]:

1. The nucleotides A and G are purines and are denoted by the symbol R, while the nucleotides C and T (U) are pyrimidines and are represented by the symbol Y.
2. The nucleotides A and C are amino and represented by the symbol M, while the nucleotides G and T (U) are keto and represented by the symbol K in the second group.
3. Finally, based on the strength of their hydrogen bonds, the nucleotides C and G have strong hydrogen bonds denoted by the symbol S, while A and T (U) have weak hydrogen bonds denoted by the symbol W.

1 $$CGR_i=0.5.(CGR_{i-1}+CGR_{s_i})\;with\;i=1,\dots,l_{S\;}and\;CGR_0=(0.5,0.5)$$

**Fig. 12:**
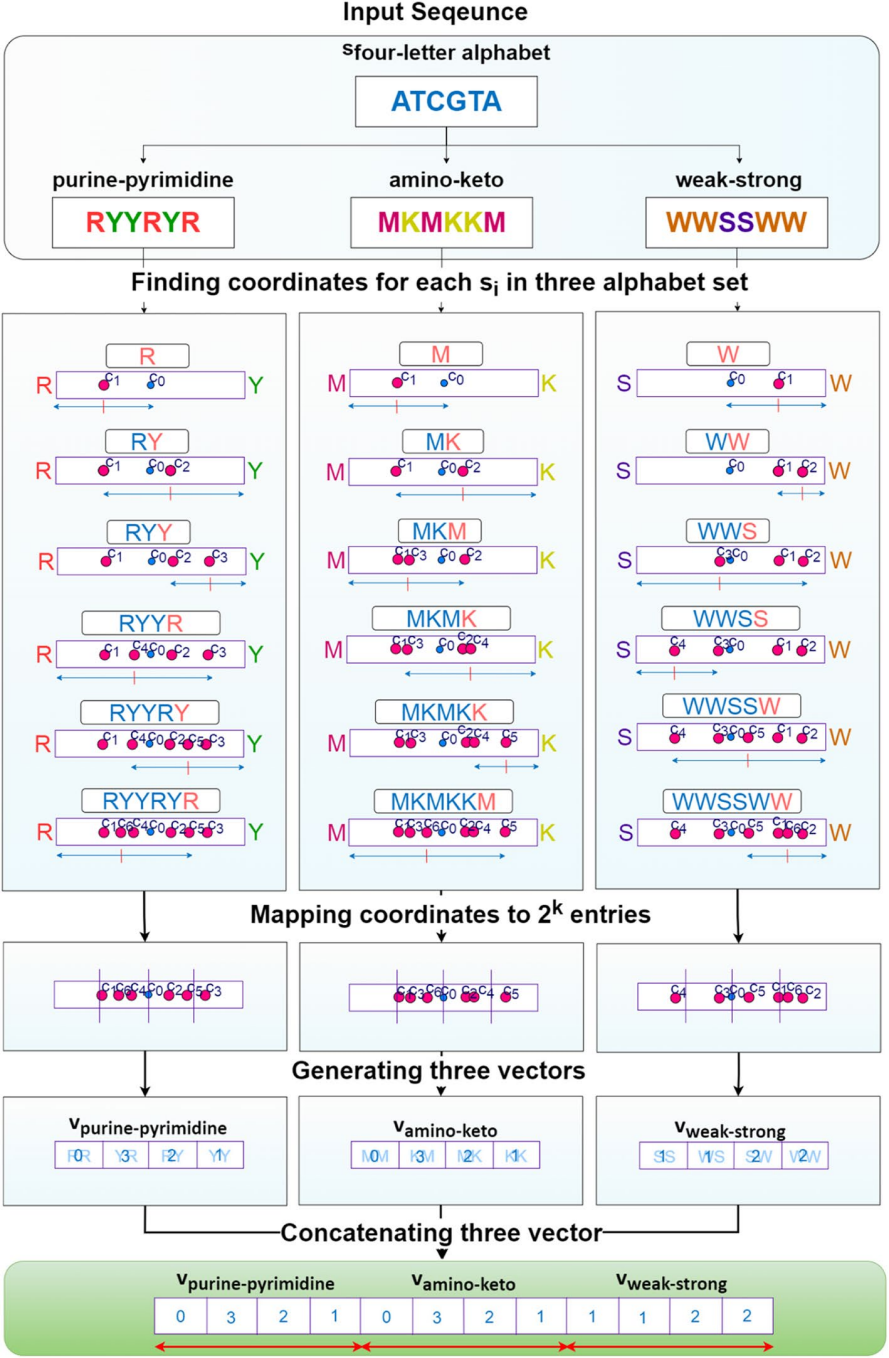
PC-mer encoding steps for sample sequence "ATCGTA"

The pseudo-code of the PC-mer method is presented in Algorithm 1. Firstly, the nucleotide symbols are converted to uppercase letters, and any unknown nucleotides are removed, based on the above categorization approach (line 1). Three 1D vectors with a length of 2^ k^, i.e., ${v}_{purine-pyrimidine}$, ${v}_{amino-keto}$, and ${v}_{weak-strong}$, are utilized to implement the PC-mer method. The two members of each set of alphabet are represented by the two corners of the corresponding vector (line 2). The PC-mer method is then applied to all nucleotides in the input sequence, as well as all three 1D vectors simultaneously. CGR_i is computed using the purine-pyrimidine property and Eq. 1, and all k-mers for each nucleotide s_i in the sequence S are counted (line 3–11). Also, the amino-keto properties (line 21–29) and strong–weak properties (line 25–35) undergo the same procedure. According to the proposed algorithm, a vector of 3 × 2^ k^ cells is constructed for each sequence (line 36), while traditional FCGR encoding produces a vector of 4^ k^ cells. As a result, our suggested encoding method, called PC-mer, significantly reduces the volume of the encoded data and computational complexity. The steps of PC-mer encoding, assuming a sample input sequence, are illustrated in Fig. 12. In the following sections, we will investigate and demonstrate the impact of this novel encoding method on the classification performance of coronavirus.

**Figure:**
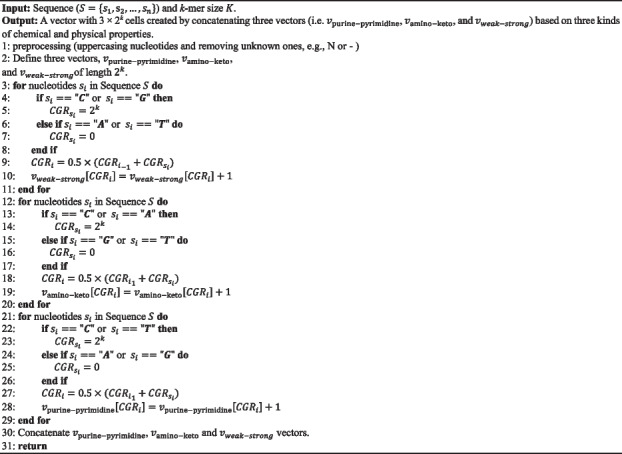
**Algorithm 1. **PC-mer based representation.

### Memory complexity

Taking memory reduction as the primary advantage of PC-mer, this section aims to analyze the memory consumption of the encoding unit in PC-mer, which can impact its functionality and execution time.

As mentioned earlier, k-mer refers to fixed-length substrings of a sequence. To extract k-mers, a sliding window of fixed length k and step size of one is applied to the sequence. This results in all possible k-mer substrings of the sequence. It should be noted that for an alphabet set of size N, there are N^k^ possible k-mers. In the case of a four-nucleotide alphabet set, there are 4^ k^ possible k-mers. Thus, the traditional k-mer representation approach has a memory usage complexity of $O\left({4}^{k}\right)$. However, as discussed in previous sections, the encoding method proposed by PC-mer utilizes three sets of two-letter alphabets. In this way, there are 2 k alternative modes for each possible k-mer, and thus, the memory complexity of PC-mer is $O\left({2}^{k}\right)$. Therefore, we can conclude that PC-mer requires 2^ k^ times less memory usage compared to traditional k-mer-based methods.

### Classification method

Classification algorithms play a crucial role in distinguishing different clusters, uncovering hidden features, and ultimately enhancing classification accuracy. While alignment-based methods can be effective for relatively small nucleotide sequences, they do not scale well for larger sequences [14, 15]. Due to their extensive computational time and complexity, it is practically challenging to analyze hundreds of whole genomes using alignment-based approaches. Furthermore, these methods may have unstable performance for highly variable regions of the genome, depending on heuristically specified parameters [13]. It is also important to note that not all datasets may meet the prerequisite assumptions of alignment-based methods, such as the homogeneity of input sequences [3, 4].

Due to their advantages and desirable characteristics, alignment-free approaches are well-suited for whole-genome comparisons [2, 18]. These methods can be classified into two main types: computational algorithms and machine learning-based approaches. Each of these types can be further divided into subclasses based on their algorithms, although there is no consensus on how to categorize them. For example, computational methods can be divided into two categories: word-based methods, which utilize k-mer frequency, and information theory-based methods, which compare informational content such as entropy between full-length sequences [8]. Machine learning-based methods can also be classified into two categories: feature-based methods, which transform genomic sequences into feature vectors for comparison, and model-based methods, which generate a model (such as a Markov model) for each sequence [8]. However, there may be some overlap in this classification, and it is not definitive.

Although various alignment-free methods are used for classification purposes, machine learning-based methods have gained more attention due to the exponential growth of sequenced data and their high potential in sequence classification. Over the past decade, deep neural networks and other machine learning algorithms have demonstrated impressive performance in data classification. However, the main challenges in using accurate classification algorithms are their computational complexity in terms of time and memory usage, the necessary preprocessing for parameter adjustment, and the requirement for developing new classifier architectures for diverse datasets. These problems are amplified by increasing the volume of input data and the number of clusters, which often occurs in biological data.

As a consequence, encoding algorithms that extract relevant features from the input data can be crucial in addressing the issues associated with most classification algorithms. With the ability to extract features using the PC-mer encoding method and create significant separation between distinct clusters, we can utilize a broad range of machine learning-based classification algorithms.

However, in this study, and without sacrificing generality, we use one of the simplest supervised classification algorithms, the Linear Support Vector Machine classifier (Linear SVM), to demonstrate the encoding capability and feature extraction of PC-mer. The main reasons for choosing this simple algorithm over other alternatives, such as convolutional neural networks or more powerful machine learning-based algorithms, are as follows:

- To mitigate the computational burden of using convolutional networks, both in terms of runtime and memory requirements, while still achieving high classification accuracy.
- To eliminate the need for adjusting numerous parameters and developing new neural network architectures.
- To enable a fair and unbiased evaluation of the encoding method regardless of the choice of classification algorithm.
- To allow for the algorithm to be run on basic computers without specialized hardware.

### Experimental setup

For evaluating the performance of the Linear SVM classifiers, we used the tenfold cross-validation approach and implemented the classifiers using the scikit-learn Python library with default settings and hyperparameters. It is important to note that all experiments, including preprocessing, training, and testing, were conducted on a desktop computer equipped with an i7-6500 2.5 GHz CPU, 8 GB RAM, and a GeForce GTX 920 M GPU with 2 GB of DDR3 RAM.

## Supplementary Information

**Additional file 1. **

**Additional file 2. **

**Additional file 3.**

## Data Availability

Source code is freely available for download at https://github.com/SAkbari93/PC-mer_Corona, implemented in python, and supported on Linux and MS Windows. The installable package of PC-mer and its document are also available at https://pypi.org/project/physicochemical-mers/.

## Abbreviations

ML: Machine Learning
PC-mer: Physicochemical k-mer
SARS-CoV: Severe Acute Respiratory Syndrome Coronavirus
SVM: Support-Vector Machine
PCA: Principal Component Analysis
