# Supplementary material for: A new profiling approach for DNA sequences based on the nucleotides' physicochemical features for accurate analysis of SARS-CoV-2 genomes
Source: BMC Genomics. 2023 May 18;24:266. doi: 10.1186/s12864-023-09373-7 (PMC10193333; doi:10.1186/s12864-023-09373-7)
Supplement: Supplementary file 1 — Additional file 1. [file 12864_2023_9373_MOESM1_ESM.docx]

A new profiling approach for DNA sequences based on the nucleotides' physicochemical features for accurate analysis of SARS-CoV-2 genomes

Saeedeh Akbari Rokn Abadi^1^ – Amirhossein Mohammadi^1^ – Somayyeh Koohi^1,*^

^1^ Department of Computer Engineering, Sharif University of Technology, Tehran, Iran

^*^ Correspondence

Contents

[Data 2](#_Toc109144481)

[Investigating the impact of size k in the PC-mer encoding method 5](#_Toc109144482)

[Confusion matrices of utilizing Linear SVM classifier and PC-mer encoding method 6](#_Toc109144483)

[References 14](#_Toc109144484)

# Data

Table S 1 Five samples from each of the 7 categories of human corona viruses for comparison (NCBI Access ID)

| Cluster | Access ID |
| --- | --- |
| HCoV-229E | AB691764  AB691766  AF304460  JQ410000  JX503060 |
| HCoV-HKU1 | AY597011  AY884001  DQ339101  DQ415896  DQ415897 |
| HCoV-NL63 | AY518894  AY567487  DQ445911  DQ445912  JQ765563 |
| HCoV-OC43 | KX538964  KX538965  KX538966  KX538967  KX538968 |
| MERS-CoV | MG987420  MG987421  MH029552  MH259485  MH259486 |
| SARS-CoV | AY545914  AY545916  AY545917  AY545918  AY545919 |
| SARS-CoV-2 | NC_045512  ON458924  ON458932  ON458934  ON458935 |

Table S 2 45 samples from SARS-CoV-2 viruses for comparison (NCBI Access ID)

| Cluster | Access ID |
| --- | --- |
| SARS-CoV-2 | NC_045512  ON458924  ON458932  ON458934  ON458935  ON458936  ON458937  ON458938  ON458939  ON458940  ON458941  ON458942  ON458943  ON458944  ON458945  ON459426  ON459428  ON459431  ON459438  ON459439  ON459440  ON459440  ON459441  ON459444  ON459445  ON459447  ON459449  ON459451  ON459452  ON459453  ON459454  ON462561  ON462562  ON462563  ON462564  ON462566  ON462567  ON462568  ON462569  ON462570  ON462571  ON462572  ON462573  ON462574  ON462575  ON462576 |

Table S 3 Specifications of 7 tests related to the [1] and dataset related to seven Human coronavirus

| Dataset | Clusters | Number of sequences | More info |
| --- | --- | --- | --- |
| Test-1:  11 families and Riboviria;  3273 sequences; | Adenoviridae  Anelloviridae  Caudovirales  Geminiviridae  Genomoviridae  Microviridae  Ortervirales  Papillomaviridae  Parvoviridae  Polydnaviridae Polyomaviridae  Riboviria | 198  126  500  500  115  102  233  369  182  304  144  500 | Maximum length: 49973  Minimum length: 2002  Median length: 7350  Mean length: 13173 |
| Test-2:  Riboviria families;  2779 sequences; | Betaflexiviridae  Bromoviridae  Caliciviridae  Coronaviridae  Flaviviridae  Peribunyaviridae Phenuiviridae  Picornaviridae  Potyviridae  Reoviridae  Rhabdoviridae  Secoviridae | 121  122  403  210  222  166  107  437  196  470  192  133 | Maximum length: 31769  Minimum length: 2005  Median length: 7488  Mean length: 8607 |
| Test-3a:  Coronaviridae;  208 sequences; | Alphacoronavirus Betacoronavirus Deltacoronavirus Gammacoronavirus | 53  126  20  9 | Maximum length: 31769  Minimum length: 9580  Median length: 29704  Mean length: 29256 |
| Test-3b:  Coronaviridae;  60 sequences; | Alphacoronavirus Betacoronavirus Deltacoronavirus | 20  20  20 | Maximum length: 31429  Minimum length: 25402  Median length: 28475  Mean length: 28187 |
| Test-4: Betacoronavirus; 124 sequences; | Embecovirus  Merbecovirus  Nobecovirus  Sarbecovirus | 49  18  10  47 | Maximum length: 31526  Minimum length: 29107  Median length: 30155  Mean length: 30300 |
| Test-5:  Betacoronavirus and  SARS-CoV-2 virus;  153 sequences; | Embecovirus  Merbecovirus  Nobecovirus  Sarbecovirus  COVID-19 virus | 49  18  10  47  29 | Maximum length: 31526  Minimum length: 29107  Median length: 29891  Mean length: 30217 |
| Test-6:  Sarbecovirus and  SARS-CoV-2 virus;  76 sequences; | Sarbecovirus  COVID-19 virus | 47  29 | Maximum length: 31526  Minimum length: 29452  Median length: 29748  Mean length: 29772 |
| Human Corona:  874 sequences; | HCoV-229E  HCoV-HKU1  HCoV-NL63  HCoV-OC43  MERS-CoV  SARS-CoV-1  SARS-CoV-2 | 76  48  71  178  200  101  200 | Maximum length: 30818  Minimum length: 20520  Median length: 29861  Mean length: 29598 |

# Investigating the impact of size k in the PC-mer encoding method

Table S 4 Investigating the impact of size k in the PC-mer encoding method generating input vectors for a Linear SVM classifier

| **k-mer** | **Metrics** | **DataSets (%)** | | | | | | | |
| --- | --- | --- | --- | --- | --- | --- | --- | --- | --- |
|  |  | **Test-1** | **Test-2** | **Test-3a** | **Test-3b** | **Test-4** | **Test-5** | **Test-6** | **SARS-CoV-2** |
| **1** | **Accuracy** | 51.29 | 75.85 | 91.80 | 98.33 | 98.33 | 96.75 | 97.32 | 100 |
|  | **F1** | 44.27 | 71.95 | 89.99 | 98.22 | 97.62 | 96.49 | 97.34 | 100 |
|  | **Precision** | 49.67 | 71.94 | 89.11 | 98.88 | 97.08 | 97.04 | 97.99 | 100 |
|  | **Recall** | 51.29 | 75.85 | 91.80 | 98.33 | 98.33 | 96.75 | 97.32 | 100 |
| **2** | **Accuracy** | 78.21 | 84.41 | 97.59 | 98.33 | 99.16 | 97.37 | 97.32 | 100 |
|  | **F1** | 77.33 | 82.50 | 97.60 | 98.22 | 98.78 | 97.35 | 97.34 | 100 |
|  | **Precision** | 79.56 | 82.68 | 98.15 | 98.88 | 98.47 | 98.03 | 97.99 | 100 |
|  | **Recall** | 78.21 | 84.41 | 97.59 | 98.33 | 99.16 | 97.37 | 97.32 | 100 |
| **3** | **Accuracy** | 88.60 | 87.54 | 97.59 | 98.33 | 98.33 | 98.08 | 98.75 | 100 |
|  | **F1** | 88.44 | 86.50 | 97.60 | 98.22 | 97.99 | 98.06 | 98.76 | 100 |
|  | **Precision** | 89.52 | 87.57 | 98.15 | 98.88 | 97.91 | 98.61 | 99.06 | 100 |
|  | **Recall** | 88.60 | 87.54 | 97.59 | 98.33 | 98.33 | 98.08 | 98.75 | 100 |
| **4** | **Accuracy** | 90.95 | 93.37 | 97.59 | 98.33 | 99.16 | 98.70 | 98.75 | 100 |
|  | **F1** | 90.87 | 93.36 | 97.60 | 98.22 | 98.78 | 98.66 | 98.76 | 100 |
|  | **Precision** | 91.51 | 93.83 | 98.15 | 98.88 | 98.47 | 99.03 | 99.06 | 100 |
|  | **Recall** | 90.95 | 93.37 | 97.59 | 98.33 | 99.16 | 98.70 | 98.75 | 100 |
| **5** | **Accuracy** | 93.70 | 94.74 | 98.07 | 100 | 99.16 | 98.70 | 98.75 | 100 |
|  | **F1** | 93.63 | 94.74 | 98.14 | 100 | 98.78 | 98.66 | 98.76 | 100 |
|  | **Precision** | 93.98 | 95.11 | 98.58 | 100 | 98.47 | 99.03 | 99.06 | 100 |
|  | **Recall** | 93.70 | 94.74 | 98.07 | 100 | 99.16 | 98.70 | 98.75 | 100 |
| **6** | **Accuracy** | 95.57 | 95.68 | 98.54 | 100 | 99.16 | 98.70 | 98.75 | 100 |
|  | **F1** | 95.50 | 95.68 | 98.63 | 100 | 98.78 | 98.66 | 98.76 | 100 |
|  | **Precision** | 95.70 | 95.95 | 99.03 | 100 | 98.47 | 99.03 | 99.06 | 100 |
|  | **Recall** | 95.57 | 95.68 | 98.54 | 100 | 99.16 | 98.70 | 98.75 | 100 |
| **7** | **Accuracy** | 97.09 | 95.89 | 98.54 | 100 | 99.16 | 98.70 | 98.75 | 100 |
|  | **F1** | 97.08 | 95.90 | 98.63 | 100 | 98.78 | 98.60 | 98.76 | 100 |
|  | **Precision** | 97.18 | 96.15 | 99.03 | 100 | 98.47 | 99.03 | 99.06 | 100 |
|  | **Recall** | 97.09 | 95.89 | 98.54 | 100 | 99.16 | 98.70 | 98.75 | 100 |
| **8** | **Accuracy** | 97.00 | 96.00 | 98.54 | 100 | 99.16 | 98.70 | 98.75 | 100 |
|  | **F1** | 96.99 | 96.00 | 98.63 | 100 | 98.78 | 98.66 | 98.76 | 100 |
|  | **Precision** | 97.07 | 96.24 | 99.03 | 100 | 98.47 | 99.03 | 99.06 | 100 |
|  | **Recall** | 97.00 | 96.00 | 98.54 | 100 | 99.16 | 98.70 | 98.75 | 100 |
| **9** | **Accuracy** | 97.28 | 96.25 | 98.54 | 100 | 99.16 | 98.70 | 98.75 | 100 |
|  | **F1** | 97.25 | 96.25 | 98.63 | 100 | 98.78 | 98.66 | 98.76 | 100 |
|  | **Precision** | 97.33 | 96.45 | 99.03 | 100 | 98.47 | 99.03 | 99.06 | 100 |
|  | **Recall** | 97.28 | 96.25 | 98.54 | 100 | 99.16 | 98.70 | 98.75 | 100 |
| **10** | **Accuracy** | 97.52 | 96.43 | 98.54 | 100 | 99.16 | 98.70 | 98.75 | 100 |
|  | **F1** | 97.51 | 96.43 | 98.63 | 100 | 98.78 | 98.66 | 98.76 | 100 |
|  | **Precision** | 97.59 | 96.63 | 99.03 | 100 | 98.47 | 99.03 | 99.06 | 100 |
|  | **Recall** | 97.52 | 96.43 | 98.54 | 100 | 99.16 | 98.70 | 98.75 | 100 |
| **11** | **Accuracy** | 97.43 | 96.11 | 98.52 | 100 | 99.16 | 98.70 | 98.75 | 100 |
|  | **F1** | 97.42 | 96.10 | 98.49 | 100 | 98.78 | 98.66 | 98.76 | 100 |
|  | **Precision** | 97.52 | 96.33 | 98.85 | 100 | 98.47 | 99.03 | 99.06 | 100 |
|  | **Recall** | 97.43 | 96.11 | 98.52 | 100 | 99.16 | 98.70 | 98.70 | 100 |
| **12** | **Accuracy** | 97.25 | 95.93 | 98.52 | 100 | 100 | 99.33 | 100 | 100 |
|  | **F1** | 97.23 | 95.90 | 98.49 | 100 | 100 | 99.36 | 100 | 100 |
|  | **Precision** | 97.38 | 96.16 | 98.85 | 100 | 100 | 99.55 | 100 | 100 |
|  | **Recall** | 97.25 | 95.93 | 98.52 | 100 | 100 | 99.33 | 100 | 100 |

# Confusion matrices of utilizing Linear SVM classifier and PC-mer encoding method


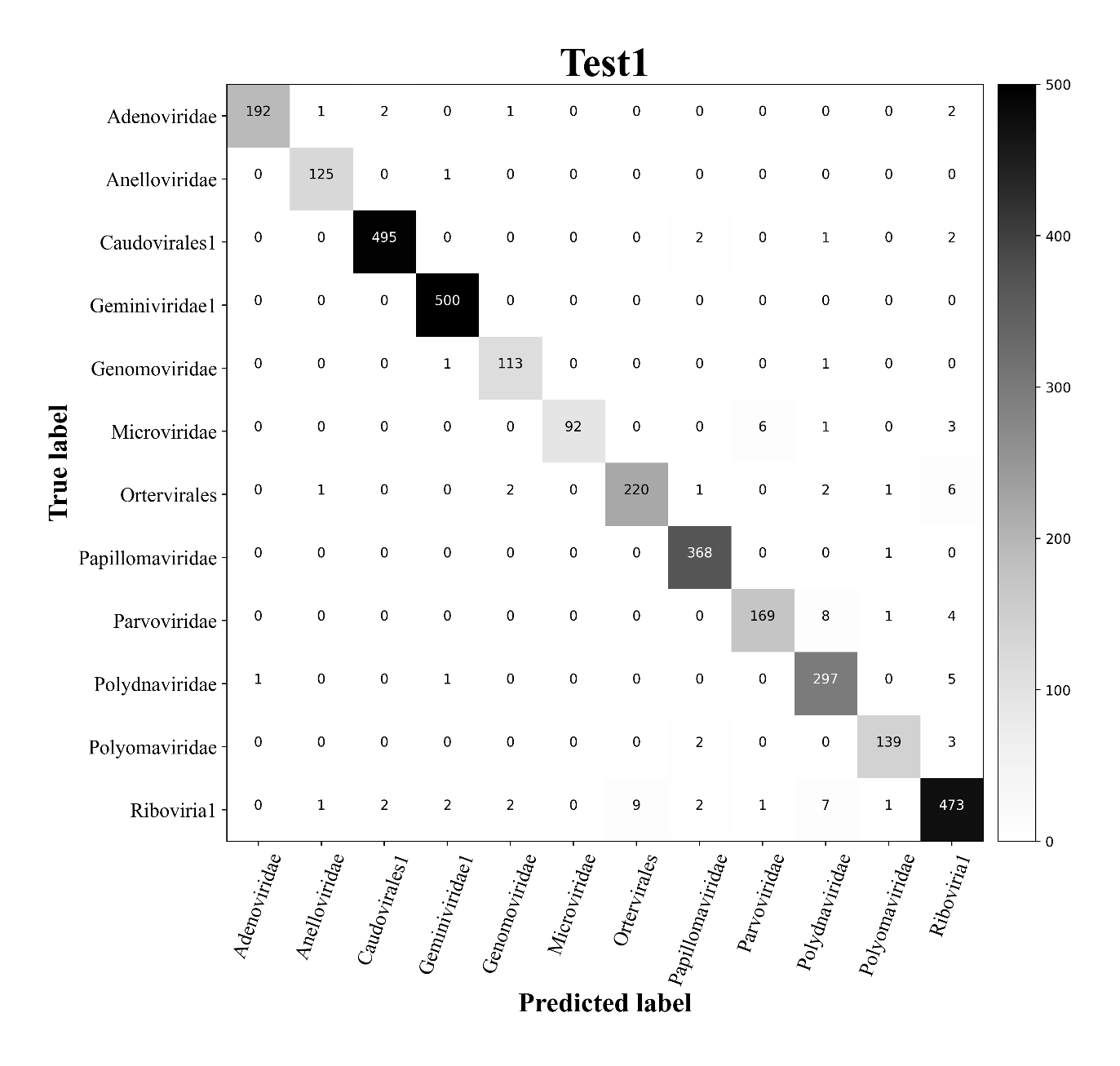


Fig. S 1 Confusion matrices of classification for Test-1 utilizing Linear SVM classifier and PC-mer encoding method


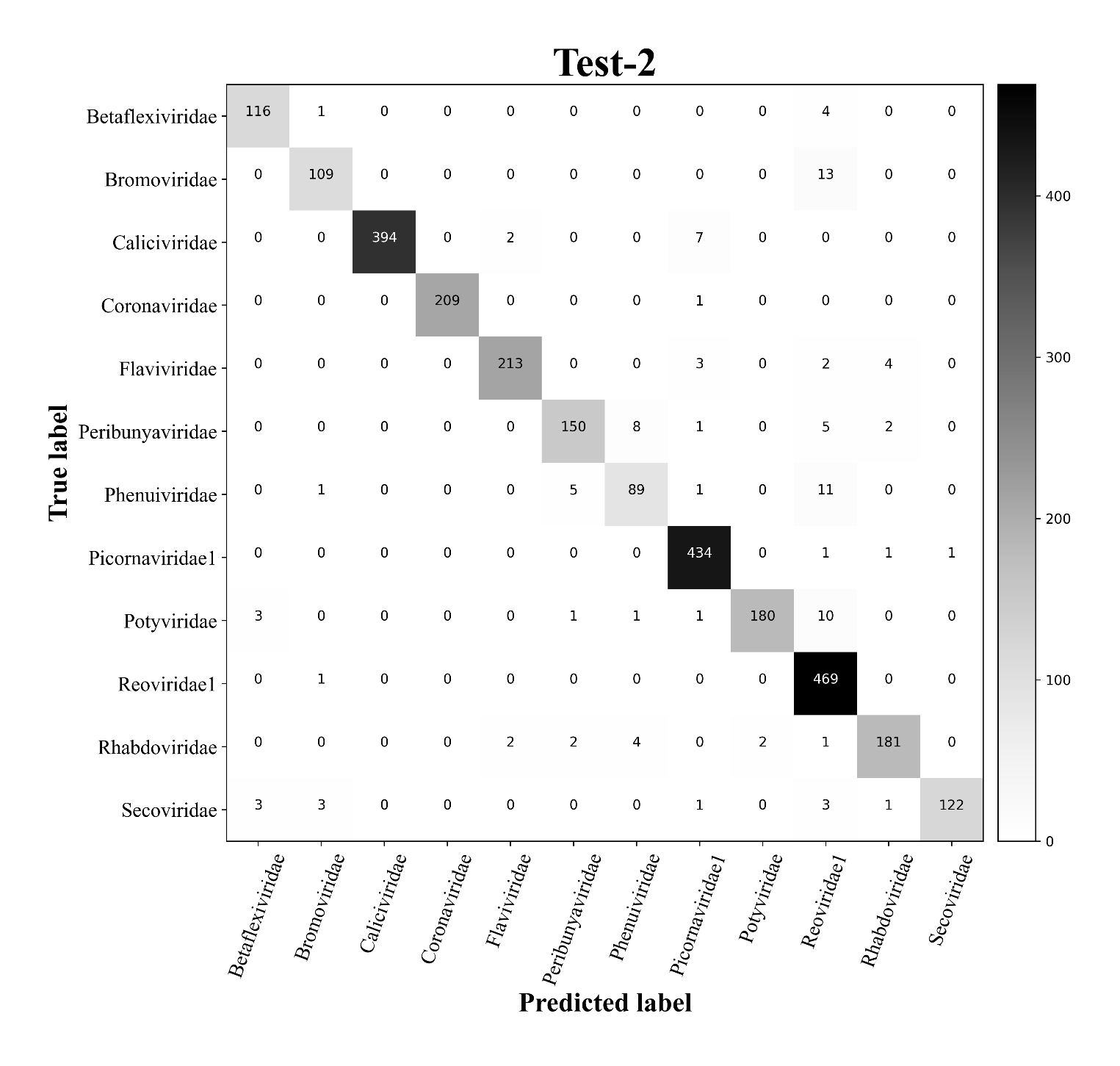


Fig. S 2 Confusion matrices of classification for Test-2 utilizing Linear SVM classifier and PC-mer encoding method


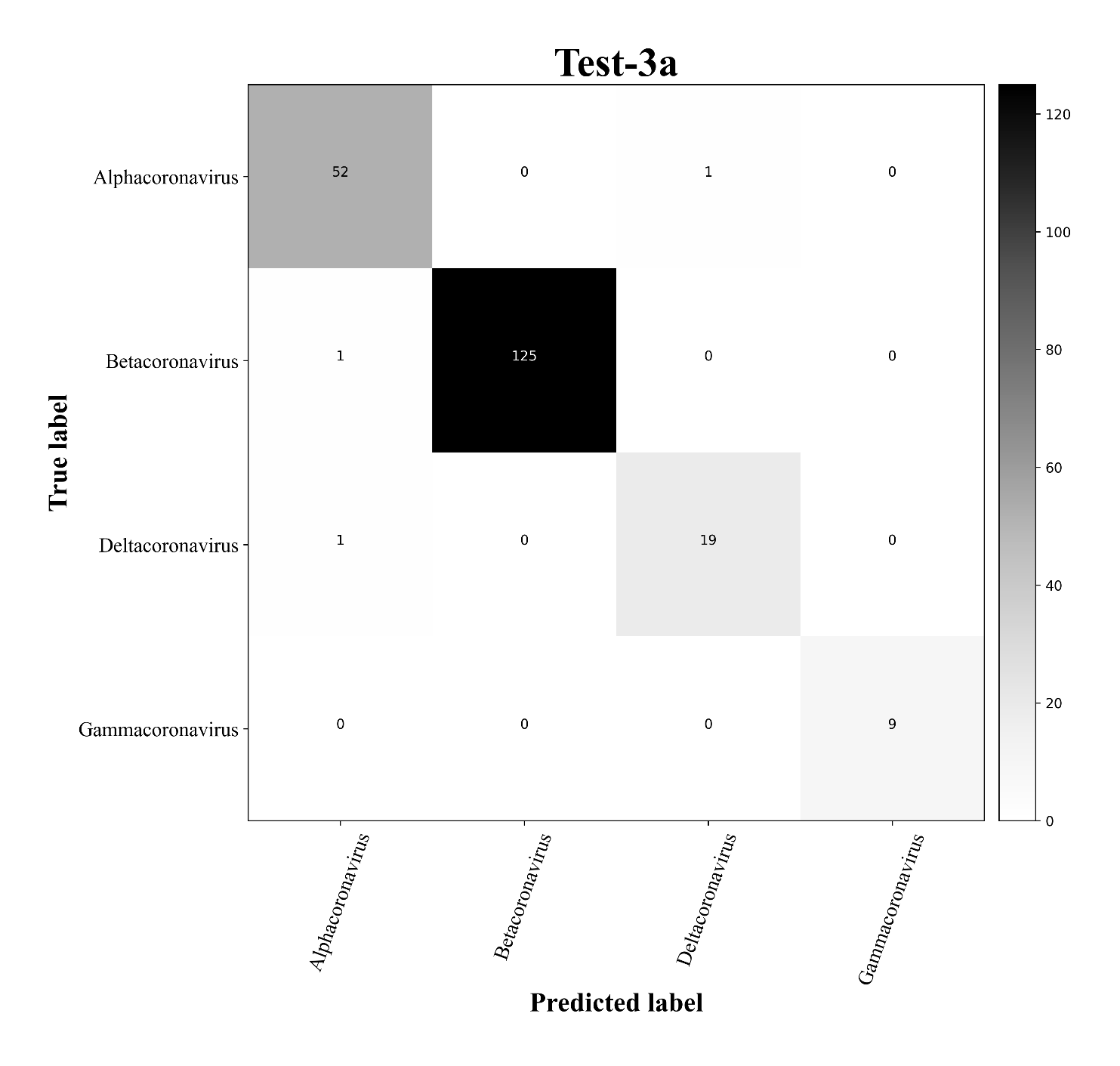


Fig. S 3 Confusion matrices of classification for Test-3a utilizing Linear SVM classifier and PC-mer encoding method


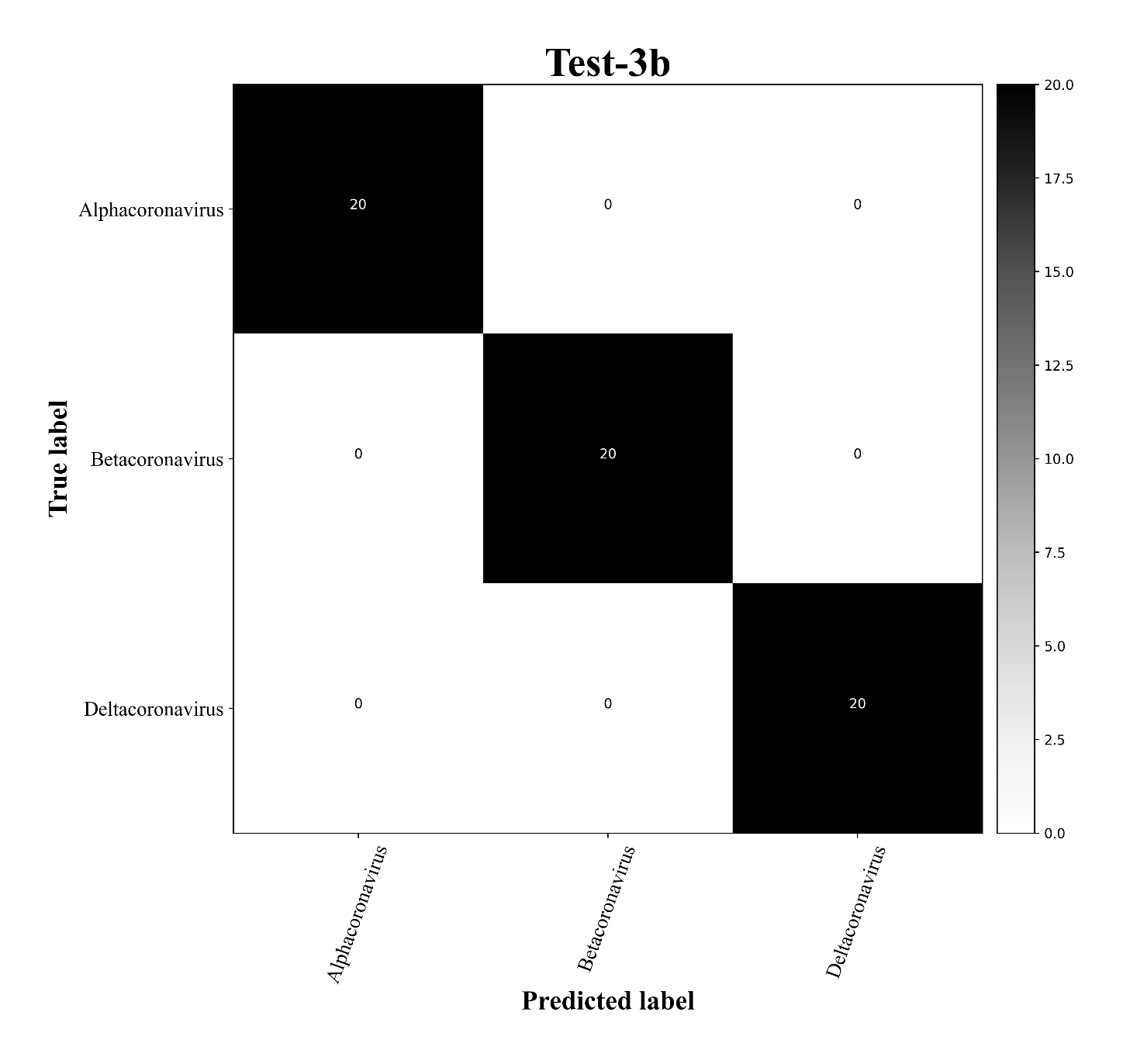


Fig. S 4 Confusion matrices of classification for Test-3b utilizing Linear SVM classifier and PC-mer encoding method


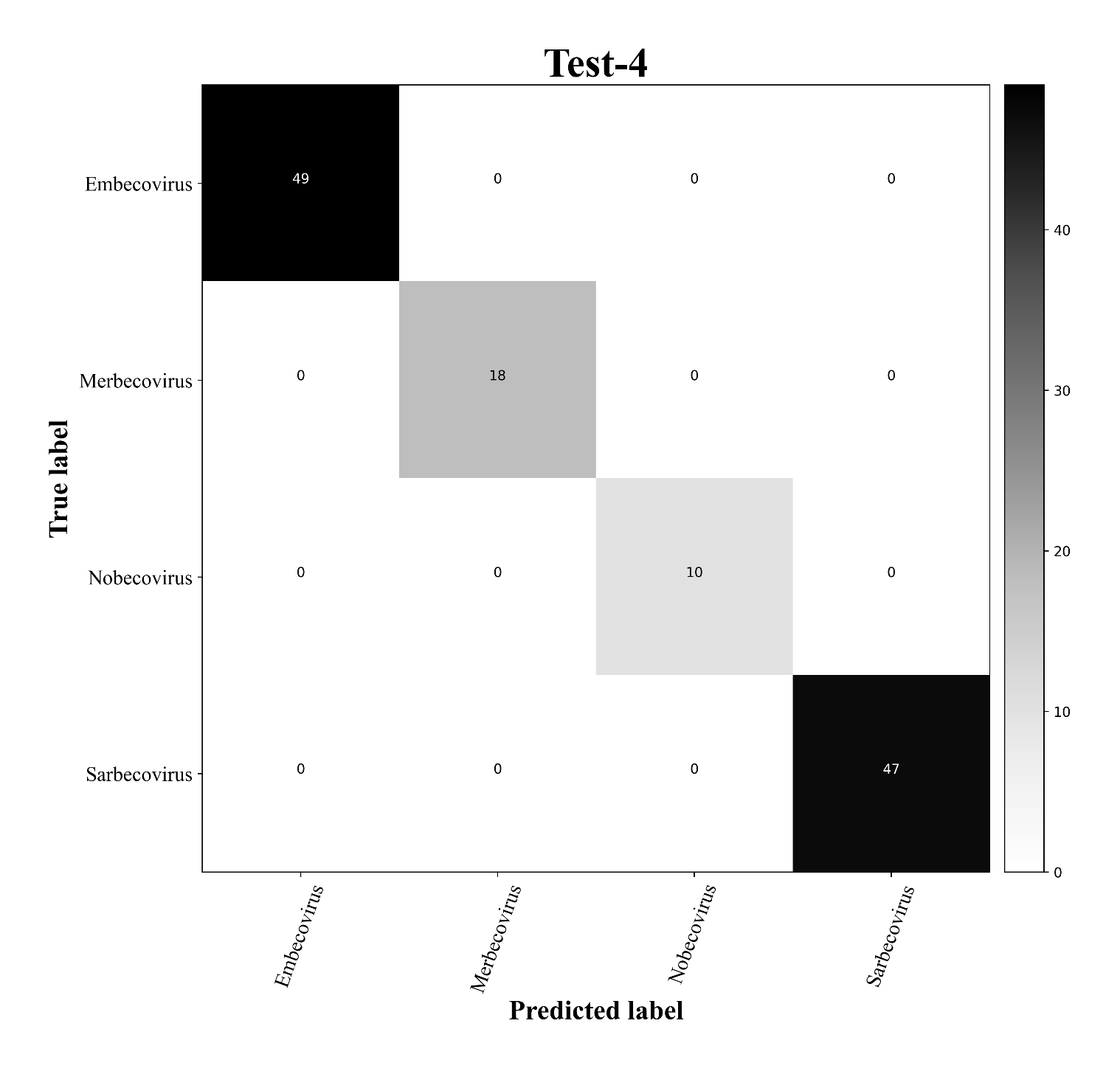


Fig. S 5 Confusion matrices of classification for Test-4 utilizing Linear SVM classifier and PC-mer encoding method


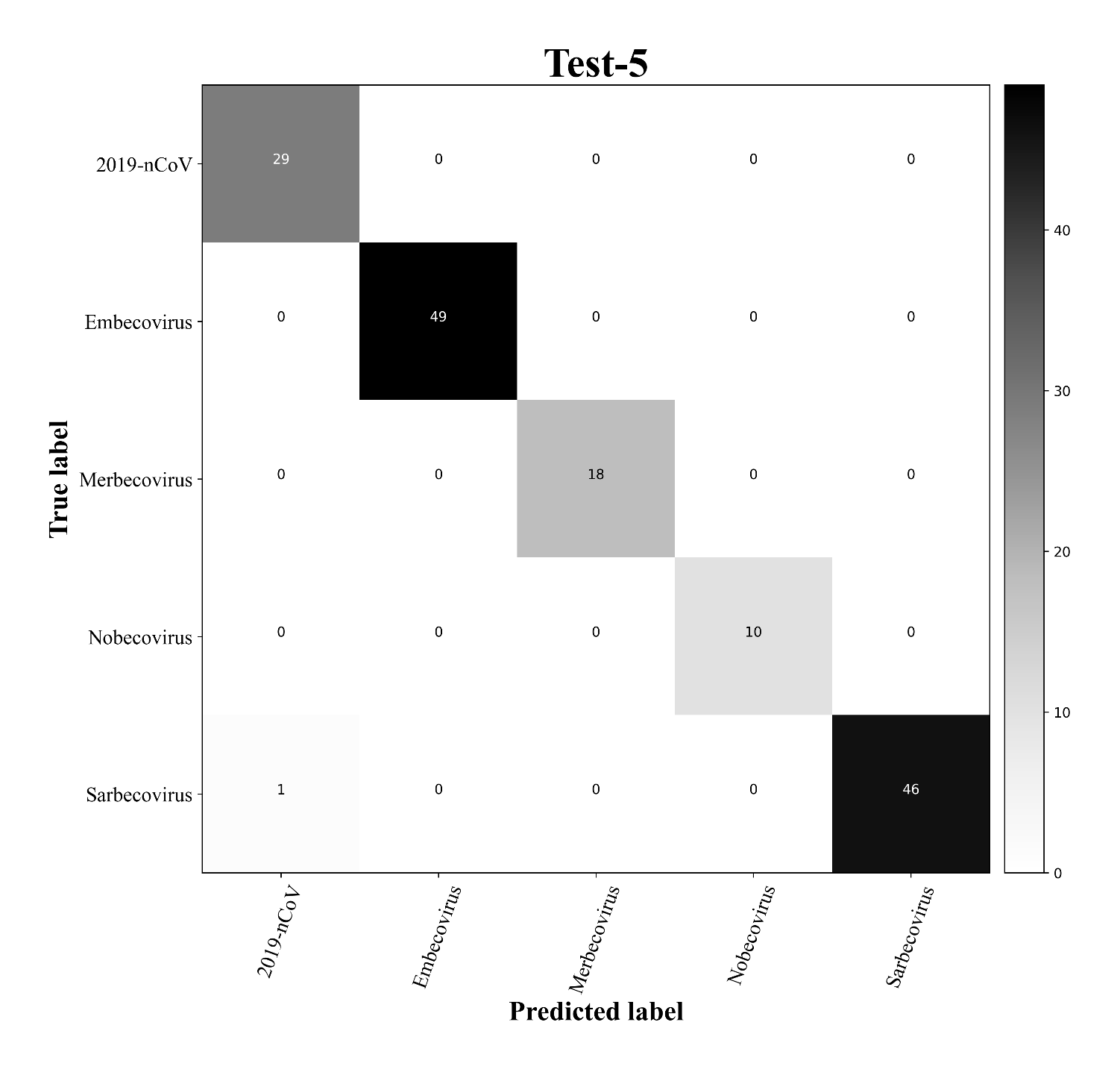


Fig. S 6 Confusion matrices of classification for Test-5 utilizing Linear SVM classifier and PC-mer encoding method


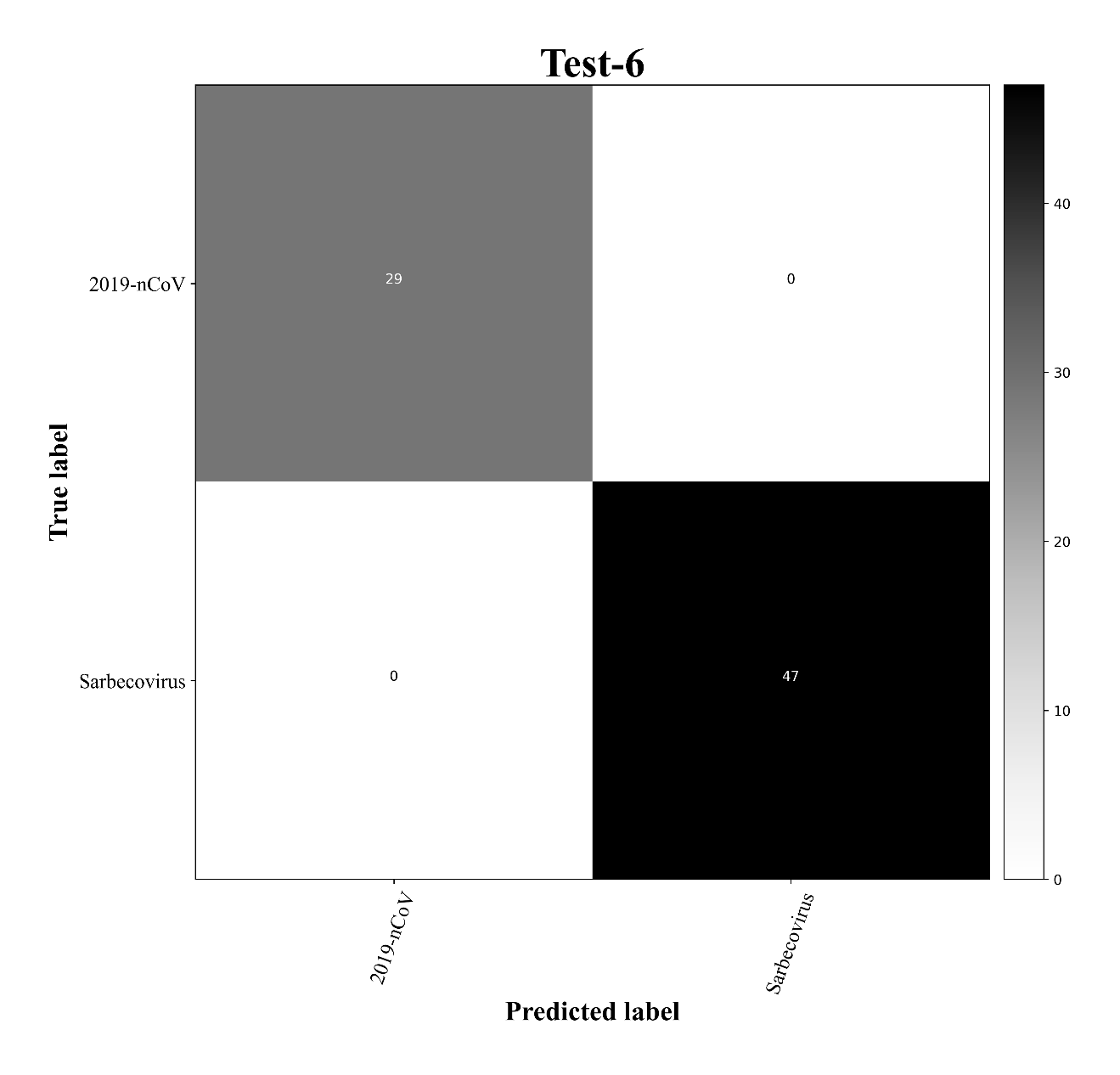


Fig. S 7 Confusion matrices of classification for Test-6 utilizing Linear SVM classifier and PC-mer encoding method


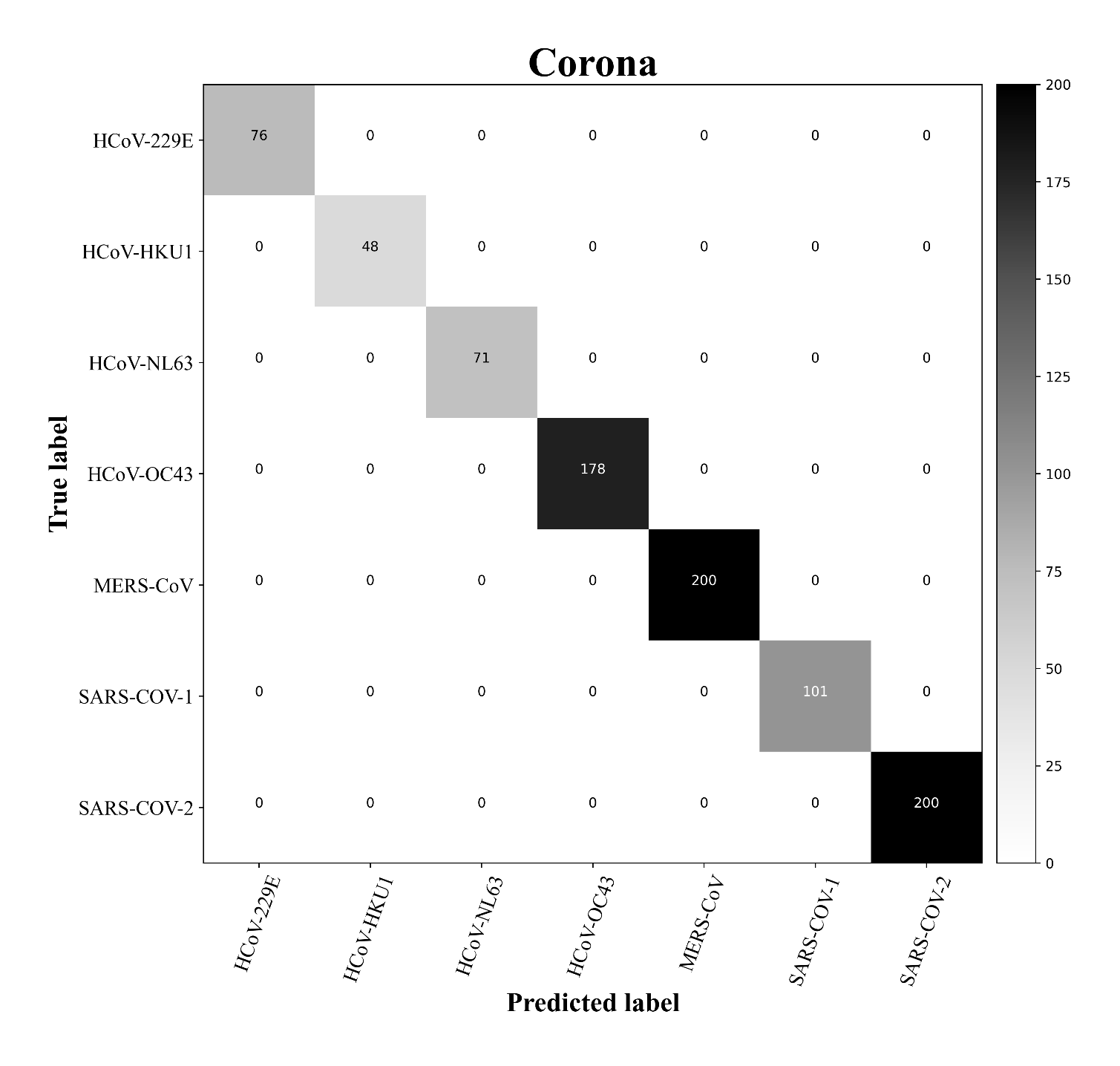


Fig. S 8 Confusion matrices of classification for Human Coronaviruses dataset utilizing Linear SVM classifier and PC-mer encoding method

# References

[1] G. S. Dlamini et al., “Classification of COVID-19 and other pathogenic sequences: A dinucleotide frequency and machine learning approach,” IEEE Access, vol. 8, pp. 195263–195273, 2020.
